# Hallmarks of Cancer Applied to Oral and Oropharyngeal Carcinogenesis: A Scoping Review of the Evidence Gaps Found in Published Systematic Reviews

**DOI:** 10.3390/cancers14153834

**Published:** 2022-08-08

**Authors:** Miguel Ángel González-Moles, Saman Warnakulasuriya, María López-Ansio, Pablo Ramos-García

**Affiliations:** 1 School of Dentistry, University of Granada, 18011 Granada, Spain; 2 Instituto de Investigación Biosanitaria ibs.GRANADA, 18012 Granada, Spain; 3 Faculty of Dentistry, Oral and Craniofacial Sciences, King’s College London, London SE1 9RT, UK; 4 WHO Collaborating for Oral Cancer, King’s College London, London SE1 9RT, UK

**Keywords:** hallmarks of cancer, biomarkers, oral cancer, oropharyngeal cancer, oral potentially malignant disorders, scoping review, systematic review, meta-analysis

## Abstract

**Simple Summary:**

This scoping review of systematic reviews aims to accurately assess the degree of existing scientific evidence on the cancer hallmarks proposed in 2011 by Hanahan and Weinberg, in the form of systematic reviews and meta-analyses, applied to oral potentially malignant disorders, oral cavity and oropharyngeal squamous cell carcinomas, in order to point out gaps in evidence and lines of research that should be implemented in the future to improve the malignant transformation prediction, diagnosis and/or prognosis of these diseases.

**Abstract:**

In 2000 and 2011, Hanahan and Weinberg published two papers in which they defined the characteristics that cells must fulfil in order to be considered neoplastic cells in all types of tumours that affect humans, which the authors called “hallmarks of cancer”. These papers have represented a milestone in our understanding of the biology of many types of cancers and have made it possible to reach high levels of scientific evidence in relation to the prognostic impact that these hallmarks have on different tumour types. However, to date, there is no study that globally analyses evidence-based knowledge on the importance of these hallmarks in oral and oropharyngeal squamous cell carcinomas. For this reason, we set out to conduct this scoping review of systematic reviews with the aim of detecting evidence gaps in relation to the relevance of the cancer hallmarks proposed by Hanahan and Weinberg in oral and oropharyngeal cancer, and oral potentially malignant disorders, and to point out future lines of research in this field.

## 1. Introduction

In 2000, Hanahan and Weinberg [1] proposed to the scientific community the idea that cancer cells should exhibit a series of distinctive characteristics or capacities that should be common to the great diversity of malignancies that affect humans and that, as a transcendent fact, would be acquired progressively throughout the multi-step process that occurs from the most incipient phases of oncogenesis, even in pre-malignant states, to those in which the cell clones exhibit all the attributes of malignancy. In this proposal for cancer hallmarks, the authors point out that tumours behave as masses of neoplastic cells whose biological activity is not exclusively limited to an uncontrolled proliferative status, as classically believed, but that they exhibit other complex capacities and interactions that transcend a simple hyperproliferative state; furthermore, the authors attribute to stromal cells an active role in tumour development and progression, collaborating in the acquisition of the distinctive characteristics of malignant cells, highlighting the importance of the tumour microenvironment in oncogenesis. Hanahan and Weinberg’s initial work was a milestone in cancer research worldwide—more than 37,000 authors to date have cited this study in their cancer research—and was an inestimable aid to the large body of evidence achieved in the years that followed. This led the authors to revise and extend the initially proposed cancer hallmarks in a new paper published in 2011 [2], which has also had an enormous impact (over 57,000 citations to date). The final proposal by Hanahan and Weinberg [2] considers a series of hallmarks (sustaining proliferative signalling, evading growth suppressors, resisting cell death, enabling replicative immortality, inducing angiogenesis, activating invasion and metastasis) that in the new paper are complemented by two enabling characteristics (genome instability and mutation, and tumour-promoting inflammation) and two emerging hallmarks (deregulating cellular energetics and avoiding immune destruction). These proposals have been studied in depth in different tumour types, although to date there is very little evidence regarding the extent to which these hallmarks of cancer apply to oral and oropharyngeal squamous cell carcinomas. The question is relevant since oral and oropharyngeal carcinomas occur with substantial frequency, demonstrating a poor prognosis in many cases, respectively, 377,713 and 98,412 new cases per year worldwide, and 177,757 and 48,143 deaths per year, (GLOBOCAN, IARC, WHO) [3]. Moreover, the considerable mortality of persons afflicted by oral cancer (50% at 5 years) has not decreased substantially in the last 40 years [4,5]. An in-depth analysis of cancer hallmarks shows that most of them are related to the acquisition of characteristics directly associated with aggressiveness of the tumours and poor prognosis of these diseases.

Thus, taking into consideration what has been said so far, we set out to carry out this study that aims to accurately assess the degree of existing scientific evidence on the cancer hallmarks proposed in 2011 by Hanahan and Weinberg, in the form of systematic reviews and meta-analyses, applied to oral and oropharyngeal cancers, and oral potentially malignant disorders, in order to point out gaps in evidence and lines of research that should be implemented in the future to improve the understanding of the biology of the tumour, malignant transformation prediction, diagnosis and/or prognosis of these diseases.

## 2. Materials and Methods

Since non-evidence-based solid knowledge has been published on the molecular biology of oral and oropharyngeal cancers and biomarker’s translational potential, a scoping review design seems pertinent to rigorously synthesize the evidence, guide future research and make recommendations on the hallmarks of oral and oropharyngeal cancers [6,7]. This scoping review adhered to the Preferred Reporting Items for Systematic reviews and Meta-Analyses extension for Scoping Reviews (PRISMA-ScR) [8].

### 2.1. Search Strategy

Cochrane Database of Systematic Reviews (aka Cochrane Library), DARE Database of Abstracts of Reviews of Effects, MEDLINE (via PubMed) and Embase databases were searched for systematic reviews published before our upper search limit date (January 2022), with no lower date limit. A comprehensive search (Appendix A) was designed considering PRESS initiative [9], conducted by combining thesaurus terms used by the databases (i.e., MeSH and EMTREE) with free terms, and built to maximize sensitivity. Hanahan and Weinberg’s biomarkers and oncogenic-related processes were identified as root keywords and sequentially extracted from a careful reading of the original paper [2]. Based on the function of these genes/proteins we catalogued them to fit Hanahan and Weinberg’s criteria. Synonyms terms of the proteins and genes included in the original Hanahan and Winberg’s paper, as well as proteins and genes with equivalent functions applicable to a particular hallmark, were also checked through HUGO Gene Nomenclature Committee, https://www.genenames.org, accessed on 1 January 2022). One challenge we encountered in the development of this research was that some oncogenesis proteins and genes have more than one function and so it is sometimes difficult to categorise them into a particular hallmark. We have tried to attribute to each protein its most relevant and best known function and therefore that has been the criterion for assigning it to a particular hallmark. We have also taken into account, when there was a protein with a multiple function, the criteria of Hanahan and Weinberg, if the authors specifically mentioned that protein, including it in the hallmark that Hanahan and Weinberg determined for it. Keywords were combined jointly with an optimal search filter designed for retrieving systematic reviews and meta-analyses (i.e., Centre for Reviews and Dissemination, CRD filter; sensitivity 99.5%, 95% CI 97.3–99.9 [10,11]). An additional final screening was performed by manually searching the reference lists of retrieved included studies and using Google Scholar. All references were managed using Mendeley v.1.19.8 (Elsevier, Amsterdam, The Netherlands); duplicate references were eliminated.

### 2.2. Eligibility Criteria

We included systematic reviews, with or without meta-analysis, evaluating Hanahan and Weinberg’s biomarkers and/or related oncogenic processes [2], in the context of oral potentially malignant disorders (classified according to the last WHO updated criteria [12]), oral or oropharyngeal cancers (i.e., malignant transformation prediction, diagnosis and/or prognosis). Oral and oropharyngeal squamous cell carcinomas were considered as distinct diseases due to differences related to their epidemiology, molecular biology, genomics and clinical presentation [4,5]. We have considered the terms “oral cancer” and “oropharyngeal cancer”, respectively, as synonyms for the terms “oral squamous cell carcinoma” and “oropharyngeal squamous cell carcinoma” for our discussion of the hallmarks of cancer. Although we are aware that oral cancer and oral squamous cell carcinoma are not absolutely equivalent terms, we have made this decision since almost the majority of these carcinomas correspond to squamous cell carcinomas. A “systematic review” was defined as a review clearly formulating a research question and using systematic and explicit methods (minimally a search strategy and eligibility criteria) to identify, select, and critically appraise relevant research, and to collect and analyse data from the studies that are included in the review [13,14]. No restrictions were applied in relation to publication language, publication date, and characteristics of the primary-level studies included in the systematic reviews (e.g., study design, geographical areas, sex and age of patients, follow-up periods, etc.).

### 2.3. Study Selection Process

Eligibility criteria were independently applied by two authors (MAGM and PRG). Articles were selected in two phases, first screening titles and abstracts for articles apparently meeting inclusion criteria, and then reading the full text of selected articles, excluding those that failed to meet the eligibility criteria. Any discrepancies were resolved by consensus.

### 2.4. Data Extraction

Two authors (MAGM and PRG) extracted data from the selected articles, completing a data collection form in a standardized manner using Excel and Word (v.16/2018, Microsoft. Redmond, WA, USA). Data were gathered on the first author, publication year, journal and JCR impact factor, study population (i.e., oral or oropharyngeal cancer and/or OPMD), sample size (i.e., number of studies), study design (i.e., systematic reviews with or without meta-analysis), biomarkers investigated and hallmarks of cancer, and key results. These datasets were additionally cross-checked in several rounds, solving discrepancies by consensus.

### 2.5. Critical Analysis and Evidence Synthesis

This scoping review was designed and developed closely following the framework of Hanahan and Weinberg’s hallmarks of cancer [2]. We examined whether the Hanahan and Weinberg’s biomarkers and oncogenic-related processes have been investigated across systematic reviews in oral and oropharyngeal cancers and OPMD clinical contexts, in order to explore the current knowledge and, synthesise the evidence. This allowed us to identify potential evidence of gaps in the knowledge. Our results are shown in descriptive tables and figures, using a systematic methodological approach, and critically discussed in depth.

## 3. Results

### 3.1. Results of the Literature Search

**Figure 1:**
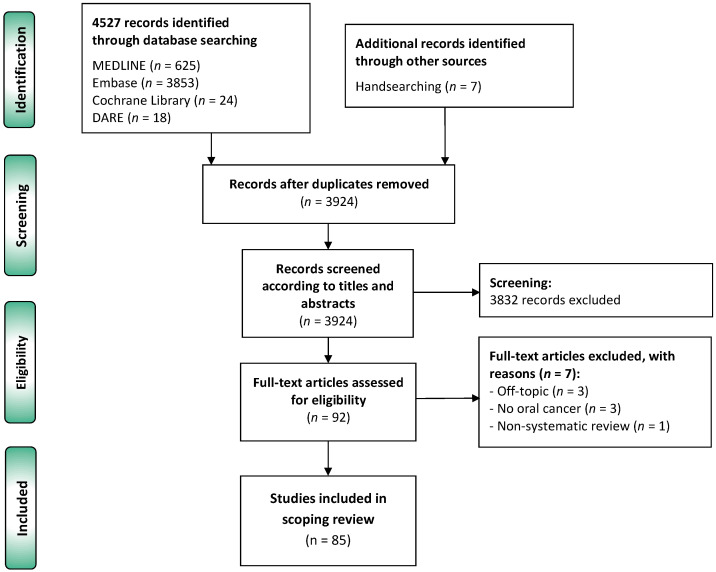
Flow diagram of the identification and selection process of the studies included in this scoping review of systematic reviews.

The flow diagram (Figure 1) depicts the results of the literature search, study identification and selection process. A total of 4534 publications were retrieved: 625 from MEDLINE (through PubMed), 3853 from Embase, 24 from Cochrane Library database of systematic reviews, 18 from DARE, and 7 by manually searching the reference lists. After duplicate elimination, 3924 records were considered potentially eligible and screened according to titles and abstracts, leaving a sample of 92 studies for full text evaluation. Finally, 85 studies meeting all eligibility criteria were included for critical analysis and evidence synthesis in our scoping review [15,16,17,18,19,20,21,22,23,24,25,26,27,28,29,30,31,32,33,34,35,36,37,38,39,40,41,42,43,44,45,46,47,48,49,50,51,52,53,54,55,56,57,58,59,60,61,62,63,64,65,66,67,68,69,70,71,72,73,74,75,76,77,78,79,80,81,82,83,84,85,86,87,88,89,90,91,92,93,94,95,96,97,98,99].

### 3.2. Study Characteristics

Table 1 summarizes the characteristics of the 85 selected studies, 8 of them analysing oral potentially malignant disorders, 70 of them oral cancer, 3 of them oropharyngeal cancer, and 4 of them both oral and oropharyngeal cancer. The first systematic review identified and included was published in 2010, and the most recent in 2022. According to the study design, all studies were secondary-level systematic reviews, and a meta-analysis was performed in most part of the studies (n = 77, 90.59%).

### 3.3. Critical Analysis and Evidence Synthesis

Table 2 summarizes the evidence derived from the research on hallmarks of oral and oropharyngeal carcinogenesis and biomarkers across secondary-level systematic reviews. Table 3 synthetizes the key results (Table 3 (A) for oral and oropharyngeal cancer, and Table 3 (B) for oral potentially malignant disorders), categorized according to the classification of Hanahan and Weinberg’s hallmarks of cancer [2]: tumour promoting inflammation was studied by 31 studies; followed by evading growth suppressors (n = 17); activating invasion and metastasis (n = 13); sustaining proliferative signalling (n = 14); deregulating cellular energetics (n = 7); angiogenesis (n = 7); avoiding immune destruction (n = 4); resisting cell death (n = 3); genome instability and mutation (n = 0) and enabling replicative immortality (n = 0).

From the previous results, a reduced sample analysed these cancer hallmarks in oropharyngeal carcinomas: evading growth suppressors (n = 5); followed by activating invasion and metastasis (n = 1); deregulating cellular energetics (n = 1), and avoiding immune destruction (n = 1).

## 4. Discussion

### 4.1. Sustaining Proliferative Signalling

A key feature of tumour cells is their ability to maintain a hyperproliferative state. Homeostatic control of epithelial proliferation in the normal oral mucosa is essential for maintaining the structure and function of the oral epithelium. This control is achieved through the influence of growth factors on their tyrosine kinase-type membrane receptors (TKRs), resulting in the downstream activation of intracellular proliferative pathways. Understanding the mechanisms of physiological regulation of proliferation in normal oral epithelial cells is hampered by the temporal and paracrine-neighbouring cell-dependent nature of growth factor production, their bioavailability being linked to their sequestration in the intercellular space and extracellular matrix. In contrast, tumour cells develop perverse mechanisms to maintain an uncontrolled hyperproliferative state which, as will be seen, acts as a driver of oncogenesis from its early stages and is essential in the acquisition of other hallmarks of cancer. Thus, malignant cells can: produce growth factors (autocrine proliferative stimulation), stimulate normal stromal cells to produce growth factors (paracrine proliferative stimulation), increase the number of growth factor receptors to make them hyperreactive to low or normal amounts of growth factors, alter the molecular structure of their growth factor receptors to enable their activation without the requirement of stimulus (constitutive receptor activation), alter the molecular structures of downstream components of intracellular proliferative pathways enabling their constitutive activation and, finally, amplify growth signals from a receptor stimulated by its ligand by transmitting them to other receptors of the same family [100,101].

**Figure 2:**
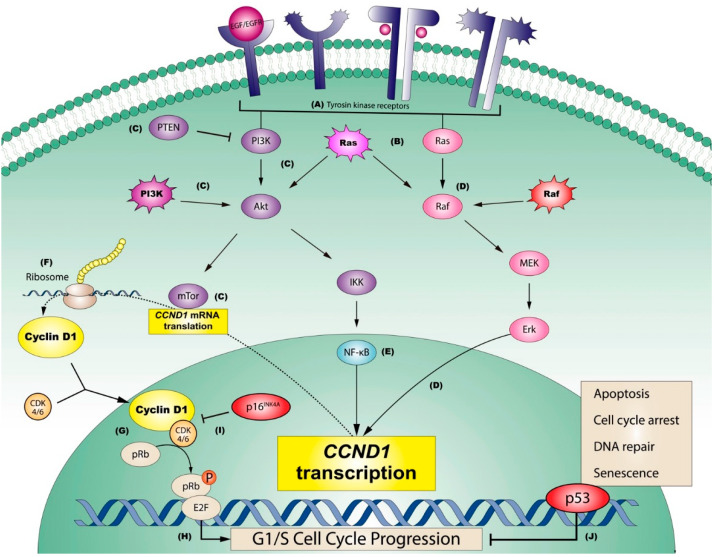
Graphic representation of the most relevant pathways regulating sustaining proliferative signalling in oral squamous cell carcinomas. (A) Tyrosine kinase receptors (e.g., EGFR or ErbB2) may be activated on the cell membrane by extracellular growth factors (e.g., EGF) or by constitutive mutations of the genes that encode them. Consequently, (B) Ras is downstream activated through the stimulation of these receptors, although Ras can be also activated by mutations, representing an influential central point in oral carcinogenesis, able to stimulate two important downstream oncogenic pathways: PI3K (pathway graphically represented in purple colour) and/or MAPK (pathway graphically represented in pink colour). (C) The PI3K pathway (PI3K-Akt-mTor, green), which can be blocked by its potent supressor PTEN (C), regulates the downstream translation of CCND1 mRNA via mTor (C). This pathway can also be constitutively activated by PI3K mutations (C). In parallel, (D) the endpoint of the MAPK pathway (Raf-MEK-Erk; in pink colour), which can also be activated by the constitutive mutation of Raf (D), is the Erk-mediated transcriptional activation of CCND1 (D). A third key pathway in the oncogenic activation of cyclin D1 is (E) NF-kB (in blue colour), which can be activated by IKK, as a consequence of PI3K pathway activation. (F) CCND1 transcriptional activation mediated by ERK, and mTor-mediated translation of its messenger RNA, are both essential for the ribosomal synthesis of cyclin D1, which forms complexes with its binding partners, CDK4/6, that finally translocate to the nucleus. (G) Nuclear Cyclin D1-CDK4/6 complexes subsequently activate the retinoblastoma pathway, in which the release of transcription factors E2F is induced by the translocation of a phosphate group, (H) with progression from G1 to S phase of the cell cycle. Activation of the retinoblastoma pathway can be prevented (I) through the potent inhibition of Cyclin D1-CDK4/6 by the product of tumour suppressor gene CDKN2A (i.e., p16INK4), blocking cell cycle advance; or alternatively by the tumour suppressor p53 (J), which plays an important role arresting the cell cycle progression, repairing the damaged DNA or finally promoting apoptosis, in an effort to prevent sustaining proliferation in cancer cells.

The epidermal growth factor receptor (EGFR or ErbB) and its ligand (EGF) family of receptors are the most important in the development of a hyperproliferative state in malignant and premalignant oral epithelial cells. This family of TKRs is composed of four types: EGFR1 (ErbB1 or HER1), -2 (ErbB2, Her2 or Neu), -3 (ErbB3 or Her3) and -4 (erbB4 or HER4). ErbBs present an evolutionarily poorly conserved extracellular domain that shows little specificity for ligand binding—different growth factors can stimulate the receptor—a transmembrane domain and a highly conserved intracellular tyrosine kinase domain. Ligand binding to the receptor induces homo—or heterodimerization of the receptor with activation of the TRK domain, phosphorylation of TRK residues in the intracellular domain of the receptor, where as a consequence, binding sites for proteins having Src homology 2 (SH2) domains and for phosphotyrosine (PTB) are established; this cascade of molecular events finally concludes with the downstream activation of intracellular proliferative pathways, essentially MAPK and PI3K/Akt, which culminate in their proliferation stimulating actions through the activation of transcription factors with stimulation of some genes that may behave as oncogenes (CCND1/cyclin D1 among the most relevant) [102] (Figure 2).

Importantly, the structural conformation of ErbB2 is particularly relevant to the acquisition of a hyperproliferative state in neoplastic cells. ErbB2 has a structure that precludes its binding to ligand—as a consequence, ErbB2 has no ligand—although this conformation, being very similar to the ligand-activated state of ErbB, enables its greater capacity to form heterodimers with other ErbB receptors; the ErbB2/ErbB3 heterodimer is the most potent in terms of activation of proliferation and transformation as it has a high capacity and potency to activate MAPK and PI3K/Akt pathways [103,104]. ErbB2 also appears to be an amplifier of stimuli transmitted by other ErbB receptors [105]. ErbB receptors are overexpressed in most solid neoplasms in humans [106], with 50–70% of lung, colon or breast carcinomas found to overexpress ErbB1 or ErbB3 [107]. Coexpression of more than one ErbB is associated with a more aggressive tumour phenotype, and coexpression of more than one growth factor is a common phenomenon in human carcinogenesis [100,101]. The most common molecular alterations of the genes encoding these receptors are amplification, N-terminal and C-terminal truncation, deletions of specific exons, tandem duplications and point mutations, among others [100,101].

Existing evidence on the involvement of ErbB receptor alterations in oral carcinogenesis is scarce and limited to three systematic reviews or meta-analyses [41,65,73], which in general present weak results due to few studies with wide confidence intervals, high levels of heterogeneity and in some cases, selection bias. The available data suggest that ErbB2 receptor overexpression is associated with poor overall survival, poor disease-specific survival and poor disease-free survival, N+ status and advanced stage [41,73]. Likewise, one study suggests that EGFR inhibition slightly improves survival compared to conservative therapy [108], and another [65] shows that ErbB mutations are extremely rare in oral oncogenesis (0.41% of oral cancer cases analysed have mutations).

#### Constitutional Activation of Proliferative Pathways

**Figure 3:**
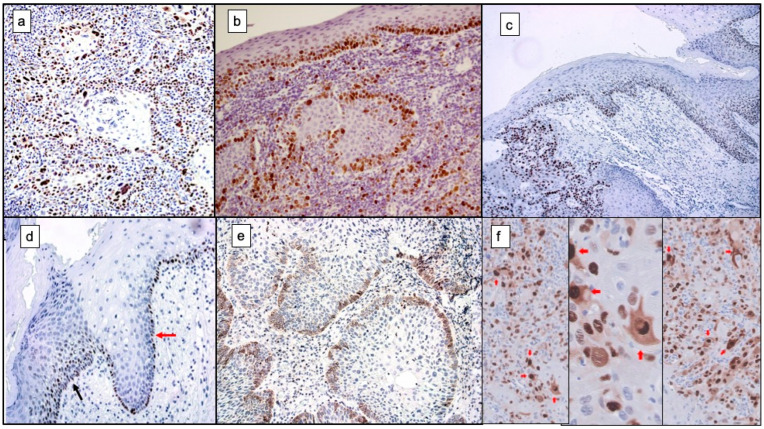
(**a**) Immunohistochemical image of cyclin D1 expression in OSCC. Note the nuclear expression of cyclin D1 at the periphery of the well-differentiated tumour nests (40× magnification). (**b**). Expression of ki67 in oral cancer and adjacent non-tumour epithelium. Note how the cellular proliferative activity of the well-differentiated tumour nests is very similar to that observed in the non-tumour oral epithelium (40× magnification). (**c**). Immunohistochemical overexpression of p53 protein in oral cancer and adjacent non-tumour epithelium. Note the early overexpression of p53 in premalignant epithelium (20× magnification). (**d**). Detail of Figure 3c. Note the overexpression of p53 in the premalignant epithelium adjacent to the carcinoma in which in the right area (red arrow) protein expression is observed in the basal layer, while in the left area (black arrow) protein overexpression is observed in the basal and suprabasal layers of the epithelium, which also shows a morphological alteration compatible with epithelial dysplasia (40× magnification). (**e**). Immunohistochemical expression of bcl-2 in the periphery of well-differentiated tumour nests (40× magnification). (**f**). Cytoplasmic cyclin D1 expression. Note that some cells show an amoeboid form of invasion probably due to the development of actin-based structures (lamellipodia and invadopodia) (40×, 200× and 40×, magnification, respectively).

Hanahan and Weinberg [2] argue in their study that somatic mutations in some components of major intracellular downstream proliferative pathways, physiologically activated by EGF/EGFR complexes, are mechanisms that are routinely established in some tumour types to achieve a sustained proliferative signal. The authors point to melanoma as an example, which we now know frequently presents mutations in the Ras protein—NRas(Q61L)—which is constitutively activated and represents a central point in the malignant transformation of melanocytes as it is capable of stimulating the two main oncogenic downstream pathways: MAPK (Ras/Raf/MEK/Erk) and PI3K (PI3K/AkT/mTOR). In melanoma, the MAPK pathway can also be activated by mutations in BRaf (BRafv600E), while the PI3K pathway can be constitutively activated by mutations in its PI3K component. As will be seen below, both pathways mediate their proliferation-stimulating activity essentially through upregulation by translational activation of the CCND1 gene encoding the cyclin D1 (CD1) protein [109,110] (Figure 3a).

MAPK pathway mutations appear mainly centred around the Ras-Raf axis suggesting that this is a hotspot for overregulation of this pathway in cancer [111]. Ras proteins are GTPases that act as switches controlling the activity of other downstream pathways. Ras mutations are found in different types of tumours and act by preventing GTP hydrolysis, allowing Ras to be permanently in an activated state that disrupts the negative feedback mechanism that attenuates proliferative signalling, exerting effects on the subsequent step in the pathway—the Raf protein. The Ras oncogene is one of the most frequently altered oncogenes in OSCC [112]. HRas mutations appear to be highly prevalent in OSCC compared to KRas and NRas mutations, having been observed in up to 55% of patients who develop OSCC [113,114], especially in the Asian continent in tobacco and betel users [115,116,117,118,119,120]. Although, as noted, there are primary-level studies on constitutive activation of MAPK pathway members in oral cancer, the absolute absence of high scientific evidence studies in the form of systematic reviews and meta-analyses in this field is striking.

The PI3K proliferative pathway is also activated in response to EGFR stimulation. Briefly, activated PI3K phosphorylates PIP2 at the plasma membrane, generating PIP3 as a second messenger, which accumulates at the plasma membrane and recruits AKT to the membrane and activates it; AKT, as a consequence, induces translation of CCND1 mRNA to encode the cell proliferation-inducing protein CD1. It should be noted that the main inhibitor of this pathway is PTEN, a tumour suppressor that dephosphorylates PIP3 and thus terminates PI3K signalling. The PI3K/Akt pathway is inappropriately activated in many human cancers as a consequence of the constitutive activation—already mentioned—of EGFRs, but also secondary to the development of molecular alterations of some components of this pathway [121]. In this sense, the first described molecular mechanism of constitutional activation of this pathway involved in oncogenesis was the loss of function of the tumour suppressor PTEN [122,123,124], although it is not clear whether this loss alone is sufficient for PI3K activation. More recently, mutations affecting p110α—the catalytic subunit of PI3K encoded by the PI3KCA gene—have been identified in up to 30% of the most common carcinomas affecting humans (breast, colon, prostate, lung and endometrial) and promote constitutive activation of the pathway [125,126,127,128]. Recent data also suggest that some cancers harbour activating mutations in p85—the PI3K regulatory subunit encoded by the PI3KR1 gene—that can probably constitutively activate this pathway by eliminating or alleviating the inhibitory effect exerted by p85 on p110 [129,130]. Genetic alterations of the three Akt isoforms (Akt-1, -2, -3) have also been observed in some cancers, including E17k mutations in Akt-1, and amplifications of the genes encoding Akt-1 and -2, and PI3KCA [131,132] have also been observed.

Closely related to the PI3K/Akt pathway are the actions of mTOR, a serine-threonine kinase that belongs to the phosphatidyl inositol family of proteins. This protein has the ability to form two complexes—mTORC1 and mTORC2—both of which are involved in the activation of cell proliferation. The proliferative activity of mTORC1 develops after stimulation by RHEB, which in turn is inhibited by the tuberous sclerosis complex (TSC1 and TSC2). Akt directly phosphorylates TSC2, inhibiting the TSC1-TSC2 complex and thereby releasing the stimulatory actions of RHEB on mTORC1. Activation of mTORC1 promotes mRNA translation of genes involved in cell proliferation, essentially MYC and CCND1/CD1. mTORC1 also inhibits 4E-BP, which in turn inhibits eIF-4E, leading to the activation of MYC and CCND1/CD1 [121,133] mRNA translation. The mTORC2 complex essentially functions by phosphorylating and activating Akt. Knowledge of the implications of mTOR in carcinogenesis comes essentially from evidence obtained from familial cancer syndromes that develop as a consequence of mutations of negative regulators of mTOR, such as TSC1-TSC2 and PTEN [133], as well as from experimental studies in mouse models of lymphoma involving alterations of eIF4E [134].

The available evidence in the form of systematic reviews and meta-analyses regarding the importance of constitutive activation of the PI3k/Akt pathway in oral cancer is scarce. A meta-analysis on all members of the pathway only reports evidence in oral cancer for Akt and mTOR (including four and one studies, respectively) [73], reporting a negative influence of their alterations on overall survival (HR ≈ 2, respectively), although the results are not very robust and with low quality of evidence [73]. Another meta-analysis including 105 publications and 8630 patients analyses the frequency of PI3kCA (13%), PTEN (4%), mTOR (3%) and Akt (2%) mutations in head and neck cancer, although data stratified by anatomical location are not reported [135].

Many of the cell proliferation-stimulating mechanisms that occur in human oncogenesis involve the upregulation of the CCND1 gene, located on chromosome band 11q13 [136], which encodes the CD1 protein; this cyclin, after complexing with its catalytic partners CDK4 and CDK6, promotes progression through the restriction point (R), which is a proliferative point of no return at the transition from G1 to S phase of the cell cycle. CD1 upregulation is closely associated with the development of some tumours including melanoma, colon cancer, breast cancer, lung cancer and OSCC [109,137,138]. It should be noted that CD1 can also exert proliferation-stimulating actions independently of CDK complex formation by interacting with transcription factors and chromatin-modifying enzymes [139] or by acting on promoter regions of gene activation [140]. Molecular mechanisms underlying CD1 overexpression in OSCC include CCND1 gene amplification—observed in 9% to 72% of OSCCs—translocations juxtaposing CCND1 with the immunoglobulin heavy chain locus [t(11;14)(q13;q32)], mutations, etc. [138]. As with other proliferation markers, there are few papers that provide high evidence in the form of systematic reviews and meta-analyses on the oncogenic and prognostic implications of CD1 in oral cancer. Our group has published two meta-analyses on the subject [49,62] which show that CD1 overexpression is significantly associated with decreased overall survival and disease-free survival, with the development of larger tumours, with an increased risk of lymph node metastasis and with higher clinical stage and histological grade. We also report that it was at the lingual site that CD1 overexpression analysis was most productive in assessing prognosis. We also report that 10% CD1 + cells was the optimal cut-off point for prognostic assessment [62]. In another meta-analysis [49] we reported that both CCND1 amplification and CD1 overexpression were significantly associated with an increased risk of progression to cancer in premalignant lesions of the head and neck and that this association was stronger for those premalignant lesions located in the oral cavity (RR = 2.31). Another meta-analytic study has reported similar results [87]. The meta-analysis by Noorlag et al. [78] also reported that both CCND1 amplification and CD1 overexpression are associated with an increased risk of lymph node metastasis. In addition, two meta-analyses [86,97] have failed to demonstrate that the AG870 polymorphism of the CCND1 gene is an increased risk factor for oral cancer.

Finally, it seems necessary to point out that advances in the understanding of the proliferative mechanisms that are established in cancer cells have led to the notion that their establishment does not always result in a maintained and perpetuated proliferative state. As acknowledged by Hanahan and Weinberg [2] “More recent research has undermined this notion, in that excessively elevated signalling by oncoproteins such as RAS, MYC, and RAF can provoke counteracting responses from cells, specifically induction of cell senescence and/or apoptosis [141,142,143]”. Furthermore, it should also be stressed that the proliferative state that develops in a neoplastic cell or group of cells is not always perpetuated over time. This has been pointed out by our research group with reference to very well-differentiated oral carcinomas that are organised in nests of neoplastic cells with very low atypicity, in which cell layers appear very similar to the normal oral epithelium. In these nests, the central areas are usually formed by keratin and the peripheral areas by proliferative neoplastic cells; this hyperproliferative state, which is lost as the tumour cells migrate to the central areas of the nest, is very similar to what occurs in normal epithelium and, as we have postulated, does not accurately reflect the potential for malignant progression of these tumours. This idea has been used by our group to justify why some pure proliferation markers, specifically Ki-67, do not accurately predict tumour prognosis [144] (Figure 3b).

### 4.2. Evading Growth Suppressors and Resisting Cell Death

These two hallmarks identified by Hanahan and Weinberg [2] take on remarkable relevance as it is imperative that tumour cells not only maintain growth-stimulating signals, but are able to evade their powerful negative growth regulation programmes and resist cell death. These actions are essentially carried out through the functions of tumour suppressor genes, the most important of which are the RB gene, which encodes the tumour suppressor protein pRb, and the TP53 gene, which encodes p53 (Figure 3c,d).

The pRb protein is frequently mutated in human cancers. RB’s physiological functions are to control proliferation by stopping the cell cycle in G1 and to promote cell differentiation and chromosomal stability [145,146], which it achieves essentially, but not exclusively, by sequestering the E2F transcription factors, thus keeping them away from their target genes. Losses of RB tumour suppressor function have a marked influence on tumour development, both in tumour initiation and in early and late tumour progression. The most representative evidence for the importance of loss of RB function in tumour initiation comes from the genetic study of members of families in which an alteration in the alleles of the RB gene predisposing to the development of familial retinoblastoma is inherited [147,148,149]. It has also been shown in cervical and oropharyngeal cancer, closely associated with HPV infection, that these viruses inactivate pRb through their E7 oncoprotein, this being the mechanism of oncogenic initiation [150,151], and similar findings have been documented for virus-induced hepatocarcinoma [152]. These tumour-initiating actions linked to the loss of RB occur both in stem cells—in which normofunctioning RB keeps them in a quiescent state, their usual situation—and in post-mitotic differentiated cells—in which the RB mutation allows them to reintegrate into the cell cycle—and, above all, in proliferative progenitor cells (called transient amplifying cells in the oral epithelium), which constitute an intermediate step between stem cells and post-mitotic differentiated cells. It seems likely that transient amplifying cells are the essential source of malignant and premalignant clones in the oral epithelium [153] where the loss of RB could maintain proliferation by preventing their exit from the cell cycle in G1, which occurs physiologically in these cells after the development of several proliferative cycles [150,151,152,153,154].

In addition to the above, some actions, in some cases paradoxical, of RB may contribute to cancer progression in both early and late phases of its evolution. This is supported by the fact that most tumour tissues only show RB alterations in the later stages of the disease [155], which probably indicates that the conserved function of RB in neoplastic cells may contribute to the development of paradoxical pro-survival actions in relation to its role as a tumour suppressor. This unexpected function is also evidenced by the demonstration that loss of RB promotes cell death [156,157], and through this mechanism, the preservation of an intact RB gene in tumour cells could prevent apoptosis and thus favour tumour cell survival. Another paradoxical function of RB is related to its ability to stimulate autophagy in neoplastic cells, a mechanism that favours their survival in the hypoxic environment in which they normally develop [158,159]. Finally, tumour progression is linked to the ability of an altered RB gene to induce undifferentiated states in mutant cells and genomic instability [160].

In oral oncogenesis, there is no secondary and tertiary evidence level, essentially in the form of systematic reviews and meta-analyses, on the prognostic and tumour developmental influence of alterations in this important tumour suppressor gene.

The TP53 gene and its product, the p53 protein, are essential in maintaining genome stability due to their ability to eliminate, through induction of apoptosis, irreparably damaged cells or to mediate DNA repair and induce cell survival in cases of moderate genome damage [161]. P53 promotes apoptosis through post-transcriptional activation of pro-apoptotic proteins, such as BAX, NOXA or PUMA [162,163] and its direct interaction in the cytoplasm and mitochondrial membrane with pro-apoptotic proteins [163]. These actions depend on the integrity of its DNA-binding domain, which explains why mutations affecting this domain inactivate the apoptotic function of this tumour suppressor protein. P53 also behaves as an inducer of senescence and differentiation—processes that prevent replication, leaving surviving, functional cells. P53 performs this function through its ability to transcriptionally activate the CDKN1A gene encoding the p21 protein, which in turn over-regulates p16INK4A, an inhibitor of CDKs that activates RB, which in turn transcriptionally induces the senescence programme [164]. P53 can also induce senescence independently of p21, via transcriptional activation of PAI1 (plasminogen activator inhibitor 1). P53 can also induce cell differentiation, which it exerts through activation of p21 and PUMA [165], which render cells resistant to reprogramming and inhibit pluripotent characteristics during cancer stem cell-induced pluripotent stem cell formation [166]. This differentiation-inducing activity appears to be relevant for the exercise of p53-mediated tumour suppression [161]. Finally, p53 plays an important role as a DNA damage repairer, which it does firstly by arresting the cell cycle with the help of p21, and secondly by activating repair genes, such as PARP1 that repairs single-strand DNA breaks, or by collaborating in the maintenance of genome stability through its centromere duplication regulatory activity [167,168,169]. It is therefore understandable that these important missions, which have earned the TP53 gene the nickname of guardian of the genome, are lost in the different ways in which the gene or its product is altered, and contribute to tumour development by allowing cells to evade growth suppressor signals or resist cell death.

To date, a significant number of systematic reviews and meta-analyses have been published on different implications of the TP53 gene and its product in carcinogenesis of the head and neck, particularly the oral cavity. Five meta-analyses have investigated the association between the rs1042522 polymorphism and susceptibility to the development of oral cancer [60,61,77,83,95]. The oncogenic mechanism linked to this polymorphism is due to the substitution of proline for arginine at codon 72, located in exon 4 of the TP53 gene. However, none of these meta-analyses has shown that this polymorphism is significantly associated with an increased risk of oral cancer. Another systematic review and meta-analysis [70] have sought to investigate the diagnostic value of circulating serological levels of p53 for oral cancer, with negative results. Another meta-analysis [99] has tried to demonstrate the potential value of p53 overexpression in head and neck cancer, but the sample of studies investigating OSCCs was also small (9 papers/413 patients). Although this meta-analysis demonstrated a significant association of p53 overexpression with worse patient survival, the effect size detected was small (HR = 1.48, 95% CI = 1.03–2.11, *p* = 0.03). A recent meta-analysis [27] has focused on investigating the frequency of TP53 gene mutations in patients with risk habits who developed HNSCC. This study reported a higher presence of mutations in smoking patients, which was statistically significant (*p* = 0.01), although the study does not allow estimating the real magnitude of this association in patients with oral cancer. Finally, our research group has published the most recent meta-analysis to date on p53 in oral cancer [16], having reported that its overexpression is significantly associated with an increased risk of malignant transformation in patients with premalignant lesions. This study presents the highest evidence to date on this biomarker, due to the sample investigated (n = 24; 1210 patients) and the significant effect size demonstrated (RR = 1.9, *p* < 0.001).

As mentioned above, many of the tumour suppressor functions of TP53 and RB are carried out through the activation of genes encoding proteins that block the cell cycle, essentially p21, p16, p27 and p15. Although p21 is one of the most important, there are no meta-analytical studies or systematic reviews that evaluate its involvement in oral oncogenesis on the basis of the evidence; something similar is also happening with p15; only one meta-analysis [96] examines the prognostic implications of p27 in OSCC, finding an association of its downregulation with poor survival, low grade, N+ status and advanced stage. P16 is a cycle inhibitor protein, activated downstream by p21, for which there is more evidence-based information in oral carcinogenesis. The etiopathogenic and prognostic implications of OSCC have been studied in five meta-analyses. Don et al. [85] reported p16 gene promoter methylation in 43% of OSCCs in their meta-analysis (15 studies), concluding that this is a frequent epigenetic mechanism whose etiopathogenic implications are still unknown. Smitha et al. [64] reported (meta-analysing 20 primary level studies) that 25.3% of OSCC cases overexpressed p16 and Mulder et al. [27] meta-analysed 12 studies reporting that immunohistochemical overexpression of p16 was more prevalent in non-smoking and non-drinking patients with oropharyngeal carcinoma, which is consistent with this tumour being more associated with HPV infection than with the classical aetiological factors of OSCC of other intraoral sites. In this regard, Ndiaye et al. [84] have also reported a prevalence of p16 overexpression in 86.7% of HPV+ oropharyngeal carcinomas. Sedghizadeh et al. [71] reported in their meta-analysis of 18 studies that p16 overexpression improved the survival of patients with oropharyngeal carcinoma 2.77-fold, which is to be expected as p16 is essentially overexpressed in HPV+ cases, which are known to behave in a more benign manner. Oropharyngeal carcinomas are currently considered a different disease from oral cavity cancer, more frequently associated with HPV-positive infection, also showing discrepancies regarding their biology [4,150]. Oropharyngeal HPV-positive tumours frequently harbour a downregulation of TP53/p53 (due to inactivation and degradation by the major viral oncoprotein E6), and exhibit increased p16 expression (due to the suppression of retinoblastoma protein [pRb] by E7, with cell cycle arrest and p16 accumulation) [4,150].

One source of growth suppressive signals comes from close contact between cohesive groups of cells. Evidence for this so-called “contact inhibition” phenomenon is obtained from the analysis of two-dimensional cell cultures grown in a monolayer, which show that close contact between these cells acts as a proliferation suppressor. It is now accepted that this mechanism also occurs in vivo and is relevant in the maintenance of tissue homeostasis. In addition, the molecular mechanisms involved in the inhibition of proliferation mediated by close cell-to-cell contact have been understood recently. One of the mechanisms involves Merlin, the product of the NF2 gene, which is considered a tumour suppressor because its deletion or mutation causes neurofibromatosis type 2, a disease in which a mutated gene is inherited in a dominant form, although there are also cases in which de novo mutations occur [170,171,172]. Neurofibromatosis type 2 is characterised by peripheral and cranial nerve schwannomas. Merlin is responsible for contact inhibition by mediating the coupling of cell surface adhesion molecules (essentially E-cadherin) to transmembrane tyrosine kinase receptors (essentially EGFR), which, while strengthening intercellular junctions, sequesters EGFRs and reduces their potential to activate intracellular proliferative pathways [173,174]. Another molecular mechanism mediating contact inhibition is exerted through the epithelial polarity protein—LKB1—which assists in the maintenance of the epithelial structure. LKB1 is now recognised as a tumour suppressor that acts through the mTOR pathway by inhibiting the activation of important proliferative oncogenes such as CCND1/CD1 and Myc [175]. In organised and quiescent epithelial structures, LKB1 is upregulated exerting its tumour suppressive actions, whereas cell dissociation involves loss of LKB1 and activation of the aforementioned oncogenes [176,177,178]. Interestingly, overnutrition may suppress LKB1-mTOR signalling which could contribute to increased cancer risk in obese and diabetic patients; conversely, activation of this pathway may account for the reduced cancer risk associated with physical exercise and calorie restriction [175]. At the moment, there is no evidence on how the loss of these tumour suppressor genes affects the risk of oral cancer development and prognosis.

TGF-β is a chemokine with a known tumour suppressor function [179] that it exerts essentially through its cytostatic, differentiation-inducing and proapoptotic effects. TGF-β signalling involves the phosphorylation of its receptor I (RTGFβI) by receptor II (RTGFβII), which in turn phosphorylates and activates the transcription factor Smad, which will form complexes with Smad4 and additional DNA-binding cofactors, complexes that will ultimately activate transcription of multiple target genes. We know that TGF-β suppresses the progression of premalignant lesions by developing a context-dependent type of response; thus, under normal conditions, loss of TGF-β function induces differentiation or cytostatic response, whereas in premalignant states, TGF-β induces apoptosis. TGF-β-mediated tumour suppressive effects can be lost during oncogenic development by alterations in TGF-β receptors (mutations or epigenetic alterations), alterations in co-receptors and ligand traps (both necessary for TGF-β presentation to its receptors), Smad4 mutations or alterations in the function of Smad4 antagonists [179,180,181,182,183]. However, as Hanahan and Weinberg [2] point out, alterations of TGF-β signalling in cancer are not only limited to its loss of function but also to the perversion of its functions; that is, tumour cells can exploit TGF-β functions to their own advantage. Thus, in many advanced-stage tumours, TGF-β signalling is diverted away from suppressing cell proliferation and instead activates a cellular programme called epithelial-mesenchymal transition (EMT), which confers cancer cells with mesenchymal cell traits, increasing their motility and favouring invasion [184,185]. In addition, TGF-β can also generate myofibroblasts in the peritumoural stroma from stromal precursor cells [186], called tumour-associated myofibroblasts, which facilitate tumour development by their ability to produce matrix metalloproteinases, cytokines (IL8) or chemokines (CXCL12). TGF-β may also contribute to the production of autocrine mitogens by tumour cells through the induction of PDGF-β [179].

In relation to oral carcinogenesis, there is very little evidence in the form of systematic reviews and meta-analyses on the influence of TGF-β on disease development or prognosis. Only one meta-analysis [48] studied the influence of certain polymorphisms of the gene encoding TGF-β on the risk of developing oral cancer, finding, in only three studies, that the 915G/C polymorphism was associated with an increased risk. 

Closely linked to the distinctive features of tumour cells regarding their ability to evade growth suppression are a number of hallmarks that characterise the resistance of neoplastic cells to cell death. Apoptosis is the main form of cell death to avoid perpetuating cell clones with irreparable DNA damage and therefore at risk of progressing to cancer, avoiding greater evils, the cell disappears without spilling its contents into the surrounding environment [141,187,188,189]. Oncogenic signalling and consequently increased proliferation are the imbalances in cell physiology that trigger apoptosis in cells on the pathway to malignant transformation. However, it has long been known that tumours are capable of outgrowing this anti-tumour response mechanism and progressing to very advanced stages [141,188]. The apoptotic machinery responds to both extracellular signals—the extrinsic pathway of apoptosis or cell death receptor pathway—and intracellular signals—the intrinsic or mitochondrial pathway. The extrinsic pathway is initiated by the production of death ligands by NK cells or macrophages (TNF, FAS-L, TLIA, TRAIL) that link to their death receptors (respectively, TNFR, FAS and TRAIL-R1 and -R2, both receptors for TRAIL). The binding of the ligand to the death receptor enables the complex to bind to procaspase 8 which, with the assistance of the FAS/FADD proteins, is activated by dimerisation and induces the activation of caspase 3, which ultimately executes apoptosis. The intrinsic apoptosis pathway is triggered by stressful intracellular situations (hypoxia, toxins, radiation, presence of ROS, etc.). This pathway involves permeabilisation of the outer mitochondrial membrane with the release of cytochrome c into the cytoplasm, which activates procaspase 9, which in turn activates caspase 3 and executes apoptosis. It should be noted that permeabilisation of the outer mitochondrial membrane is regulated by proteins of the BCL-2 family. Some of them, such as Bax (Bcl-2-associated protein X) and Bak (Bcl-2 homologous killer antagonist), are pro-apoptotic factors necessary for the formation of pores in the mitochondrial membrane, which in turn are regulated by anti-apoptotic proteins whose archetype is Bcl-2 (Figure 3e), together with its close relatives Bcl-xL, Bcl-w, Mcl-1 and A1 that bind and inactivate Bax and Bak (Figure 3e) [188]. Finally, “BH3-only” proteins act by interfering with anti-apoptotic Bcl-2 proteins or directly stimulating pro-apoptotic members of this family [188,190]. The essential trigger of apoptosis in response to irreparable DNA damage is the tumour suppressor gene TP53 [162,191] which acts by up-regulating the proapoptotic proteins Bax, Noxa, PUMA and BH3-only, through post-transcriptional activation of the genes encoding them. Finally, apoptosis can also develop through the actions of the Bim protein. Clearly, human oncogenesis and tumour progression require that this essential mechanism for eliminating damaged cells is inactivated. Tumour cells can inactivate apoptosis through several processes. Of particular relevance is the loss of tumour suppressor functions of the TP53 gene, although neoplasms also achieve this goal by upregulating anti-apoptotic proteins (Bcl-2, Bcl-xL) or downregulating pro-apoptotic factors (Bax, Bim, Puma), among other mechanisms.

Autophagy represents another mechanism by which the cell responds to a stressful situation essentially related to the lack of nutrients [192]. Through autophagy, cell organelles can be broken down and the resulting catabolites reused for biosynthesis and cellular energy metabolism. It is a process that requires the assistance of autophagosomes—vesicles that bring cell organelles into contact with lysosomes for degradation. The molecular mechanisms involved in autophagy essentially involve the beclin-1 protein, which belongs to the “BH3-only” protein superfamily and is bound to the Bcl-2/Bcl-xL proteins. In situations of cellular stress, beclin-1 unbinds from Bcl-2/Bcl-xL and is free to trigger autophagy. Likewise, the PI3K/Akt/mTOR pathway, activated by survival signals, inhibits autophagy [191]. Autophagy has been linked to cell death in senescent cells and to the destruction of cancer cells [193] and is therefore considered to be a tumour suppressor mechanism [194]. Paradoxically, however, it has also been observed that pharmacological inhibition of autophagy with chloroquine stimulates rather than inhibits cancer cell death. It seems therefore that autophagy could represent an adaptive mechanism of neoplastic cells against stress linked to the hypoxic environment in which they usually develop [192].

Necrosis is another type of cell death that differs from apoptosis and autophagy in that the cell collapses and releases its contents into the microenvironment; this generates stimuli that recruit inflammatory cells of the immune system [195,196,197]. The physiological function of these immune cells is the clearance of necrotic debris from the tissue microenvironment, although there are now consistent lines of evidence that tumour cells can benefit from tumour-promoting activity linked to the inflammatory infiltrate, both at the early stages of oncogenesis and in later stages of invasion and during metastatic development [197]. We now know that this is not a strictly random process linked to the establishment of severe and sudden cellular damage—radiation, heat, chemical aggression, etc., but that in some circumstances it could be under genetic control aimed at obtaining oncogenic advantages [195,198].

### 4.3. Enabling Replicative Immortality

Human cells develop according to a limited survival programme that is essential to allow their progeny to occupy their space—a necessarily limited space in the organism—and perform the functions for which they were created with maximum efficiency and minimum risk. This limited survival programme is associated with a physiological limitation in their proliferative capacity, which does not go beyond a restricted number of cell division cycles. In contrast, neoplastic cells must acquire the ability to overcome this limited proliferation in order to immortalise and persist pathologically in the organism, which is a hallmark of cancer of great relevance [2]. The limitation of the physiological survival of normal cells is related to the functions of fragments of the genome called telomeres. These are tandem hexanucleotide sequence repeats (TTAGGGs) that are located at the ends of chromosomes with the essential mission of protecting them [199]. Experiments with cultured human fibroblasts [200] have shown that they have a limited replicative potential (60–80 cycles) and that each cycle results in a shortening of their telomeres. In the late life stages of fibroblasts in culture, extreme telomere shortening and deterioration induces an irreversible cellular state called senescence, which results in viable quiescent cells, and in the case of cells that are able to circumvent this senescence programme, a second barrier to survival called crisis is established, involving cell death [201]. The progressive shortening of telomeres and their extreme deterioration allow chromosomal aberrations to develop, including chromosome breakage and fusion bridges (BFB), fusion of chromosome ends-end-to-end fusion, generating unstable dicentric chromosomes and translocations between non-homologous chromosomes (non-reciprocal translocations), involving amplifications and deletions of areas of the genome harbouring oncogenes and tumour suppressor genes, triggering a TP53 gene response that ultimately results in the establishment of senescence or crisis programmes [202,203].

According to the above, most incipient neoplasms will be eliminated as a consequence of the development of senescence or crisis programmes linked to the normal function of TP53, and on the contrary, some incipient neoplasms will overcome these senescence and crisis programmes and develop clones of immortalised cells; for this, it seems necessary, as we shall see, that the normal function of TP53 is lost and also that the length of its telomeres is maintained [2], for which the help of a DNA polymerase enzyme, called telomerase, which adds hexanucleotide segments to the telomeres, maintaining their length, is decisive. Telomerase is overexpressed in most immortalised cells and in cancer cells, and its increased activity confers resistance to senescence and crisis/apoptosis. The idea that alterations in TP53 and the level of telomerase activity are essential for inducing replicative immortality in tumour cells has been supported by animal experimental studies that have shown that mutant mice lacking TP53 function and telomerase activity develop telomere shortening with BFB, cell survival and high genome mutability [1,2,199]. Furthermore, the study of tumour tissue, particularly breast cancer, has shown that tumour cells can perversely modulate their telomerase activity to their own advantage in order to immortalise themselves. Thus, in pre-invasive breast lesions, FISH has documented the existence of short telomeres, low telomerase activity and non-clonal chromosomal aberrations resulting from the existence of chromosomal instability due to the loss of telomeric protection; the alterations are not clonal because in the pre-invasive state there has not been enough time for the clonal expansion of the mutated cells. In this situation, if TP53 is functioning properly, this increased oncogenic signalling will trigger its response and induce crisis/apoptosis, eliminating most of these incipient neoplasms. Conversely, in the absence of TP53 function, pre-invasive clones will not be subject to the tumour suppressive control exerted by the TP53 gene. At this point it is also relevant to observe what happens in advanced breast carcinomas; these show high levels of telomerase activity, elongated telomeres, immortalised cells and clonal genomic alterations; here, the ability of tumour cells to overexpress telomerase at later stages of oncogenesis has allowed tumour cells to survive and expand clonally, while the lack of telomerase activity at early stages of oncogenesis allows mutations linked to telomere shortening and chromosomal instability to be acquired [204,205].

We should note with surprise that this attractive mechanism of cell survival and promotion of mutagenesis linked to telomerase activity has not received any attention in the field of oral carcinogenesis and there are no studies on the subject with evidence-based conclusions.

### 4.4. Induction of Angiogenesis

As caricatures of normal tissue, tumours must also be endowed with a vascular network that allows for the supply of nutrients and oxygen, and the removal of catabolites and CO_2_ resulting from cell metabolism. Sustained neoplastic growth requires the neoformation of tumour vasculature, in a process called neoangiogenesis, which is finely regulated by stimuli involving the same mechanisms that occur in normal tissue during the physiological processes required for the formation of new blood vessels—embryogenesis, wound healing, female reproductive cycle, etc. Human carcinogenesis is associated with the development of new tumour vessels from very early periods of malignant transformation, even in pre-invasive dysplastic stages [206], which consolidates neoangiogenesis as a hallmark of cancer.

The tumour neoangiogenic process is regulated by a switch that is activated or inactivated by factors—usually proteins—that stimulate or inhibit angiogenesis [207]. The activating prototype of the angiogenic switch is constituted by the vascular endothelial growth factor (VEGF) family, the most relevant member of which is VEGF-A; they are ligands that bind to tyrosine kinase receptors (VEGFR-1, -2, -3) and induce neoangiogenesis. VEGF production can be autocrine (by endothelial cells in blood vessels) or paracrine (by tumour or stromal cells). VEGF signalling from tumour cells can originate from the activation of common cancer oncogenes—Myc, Ras—or from mutations and polymorphisms of the VEGF gene. Other proangiogenic factors that have been shown to be important in tumour neoangiogenesis are members of the fibroblast growth factor (FGF) family; they exert their function by stimulating FGFR receptors on endothelial cells and inducing them to release other angiogenic factors—angiopoietin 2, VEGF-β, hedgehog. Interestingly, paracrine proangiogenic signalling may also come from inflammatory cells infiltrating the tumour and pre-invasive lesions, or found in the peritumoural stroma (macrophages, neutrophils, mast cells, etc.,) [208,209].

The switch that regulates angiogenesis is also subject to inhibitory stimuli mediated essentially by thrombospandin 1 (TSP-1), plasmin (angiostatin) and collagen type 18 (endostatin); these inhibitors behave as barriers that limit neoplastic expansion. Finally, pericytes, which cover endothelial cells, until recently considered passive observers of the tumour neoangiogenesis process, are now recognised as relevant in the maintenance of functional tumour vasculature [210].

Although the above represents the most relevant molecular events associated with tumour angiogenic regulation, some effects mediated by other pathways also play a role. For example, it is known that endothelial cell-mediated PDGF-β signalling attracts pericytes to the new vessel and that inhibition of the PDGF-β receptor decreases tumour growth by causing pericyte detachment [211]. Likewise, the actions of angiopoietin 1 and 2, NOTCH and WNT signalling also stimulate tumour angiogenesis [211].

As with other hallmarks, evidence-based results on the implications of the study of neoangiogenesis on the prognosis and risk of oral cancer development are scarce. Most of them refer to the role of VEGF. One meta-analysis [94] found based on the study of nine primary level papers that immunohistochemical overexpression of VEGF was significantly associated with poor survival. Three meta-analyses have investigated the influence of various types of VEGF gene polymorphisms on the risk of developing oral cancer [76,92,93], only finding an increased risk associated with the 936C/T polymorphism. Finally, a systematic review without meta-analysis in head and neck carcinomas [69] found in the subgroup of studies on oral cancer (two studies) a lack of influence of FGFR overexpression on survival.

### 4.5. Activation of Invasion and Metastasis

The development of distant organs and lymph node metastasis is an essential cancer process that dramatically conditions the prognosis of many tumour types, including oral cancer. There is evidence that metastatic spread can be a very early phenomenon in carcinogenesis, even in apparently non-invasive premalignant lesions [212], which highlights the importance of this aspect of tumour cell biology and reinforces its consideration as a hallmark of cancer.

The metastatic process unfolds in a series of orderly phases that include invasion—whereby transformed cells leave the tissue of origin to infiltrate adjacent tissues—intravasation of malignant cells into blood and lymphatic vessels, transit through the vascular tree, extravasation, establishment of micrometastases, and finally colonisation, which involves the growth of micrometastases into macroscopically evident tumours [213]. In each of the phases of the metastatic process, diverse and complex molecular mechanisms develop that require, as we will see, interactions with the microenvironment in which the primary tumour develops and especially with the microenvironment of the tissue that receives the metastasis.

The invasive phase is governed by a regulatory programme called epithelial-mesenchymal transition (EMT), whereby epithelial cells change from polygonal epithelial morphology to spindle mesenchymal morphology, increase their motility, lose expression of typical epithelial markers—essentially E-cadherin—express mesenchymal markers—vimentin—and become resistant to apoptosis [214]. A determining aspect in the EMT programme is the loss of E-cadherin expression, an adhesion molecule essential for holding epithelial cells together in clusters that hinder their motility. The EMT phenomenon involves the dissociation of transformed cells and their capacitation for invasion and metastatic spread. EMT is therefore considered to be the driving force for the spread of epithelial neoplasms. A relevant fact of the EMT phenomenon is its reversible character so that through the opposite phenomenon, called epithelial-mesenchymal transition (MET), transformed cells can reacquire an epithelial phenotype with re-expression of E-cadherin and recovery of their intercellular junctions, which will be beneficial for the growth of metastatic colonies. The EMT phenomenon can also be partial or hybrid [214], with cells developing mesenchymal and epithelial phenotypes, termed metastables, which translates into a flexible and dynamic cellular state, which develops to reverse—ultimately control—the EMT phenomenon [215,216,217,218]. The mechanisms by which EMT is established are essentially due to the actions of certain transcription factors (Snail, Slug, Twist, zeb1/2), knowledge of which derives from embryogenesis; these transcription factors activate genes responsible for the events that unfold in epithelial cells during EMT, notably the loss of E-cadherin expression [219]. Another important aspect of EMT is its heterogeneous character in tumour tissue, being frequently observed at the margins of invasion of carcinomas, while the central areas retain a purely epithelial morphology with the preservation of their intercellular adhesions. This fact probably reflects that stimuli from the peritumoural stroma [220] are a driving force for EMT, and there are some lines of evidence in this direction [221].

The invasive phase takes on different morphological appearances that are probably due to the activation of different molecular programmes. Mesenchymal-like” invasiveness involves the establishment of EMT with dissociation of tumour cells. In “collective” invasiveness, cohesive groups of transformed epithelial cells invade the peritumoural tissue. Finally, in the so-called “amoeboid” form of invasion, tumour cells show morphological plasticity that allows them, through the development of actin-based protrusive structures—lamellipodia and invadopodia (Figure 3f)—to take advantage of tissue interstices to invade [2,222]. The molecular mechanisms involved in invasion in the form of cohesive cell clusters or amoeboid-like invasion are not well understood, nor to what extent EMT is involved in their development, although our research group has reported that cytoplasmic overexpression of cyclin D1 is associated with the acquisition of amoeboid morphology by invasive cells in oral carcinomas (Figure 3f) [223].

Colonisation, the final stage of the metastatic process, enables macroscopically evident tumour growths to develop in the tissues harbouring the metastases; this process, which requires the successful growth of micrometastases, is probably the most complex, as evidenced by the fact that many patients develop multiple micrometastases that successfully spread but fail to colonise the tissue in which they implant [213]. A surprising fact in the biopathology of micrometastases refers to the ability of some tumour types to keep these small neoplastic seedings silent—latent—for very long periods of time. It has been suggested that some tumours may release systemic factors that suppress the growth of micrometastases, causing them to remain dormant; this idea is supported by the observation of massive metastatic growths after the removal of the primary tumour [224]. Other tumours, however, develop metastases years after resection of the primary tumour, reflecting not a true period of induced latency, but the establishment of multiple colonisation attempts until the acquisition of a successful growth mechanism [225]. Micrometastases may find it remarkably difficult to grow in relation to deficient nutritional microenvironments. In such cases, autophagy mechanisms have been found to establish viable latency states from which metastatic cells can emerge when nutritional conditions improve [226]. Finally, colonisation may also be hindered by the inability of micrometastases to develop neovascularisation to support the vigorous growth required for colonization [227] or by growth limitations imposed by the anti-tumour immune response [228].

Existing evidence on the activation of invasion and metastasis as a hallmark of cancer in oral carcinogenesis is limited to systematic reviews and meta-analyses on E-cadherin and on transcription factors involved in the development of EMT. Five systematic reviews and meta-analyses address the prognostic value of E-cadherin downregulation and loss of E-cadherin expression in head and neck cancer [98,229,230], oral cancer [82] and in oropharyngeal carcinoma [25]. They report that loss of E-cadherin expression is a marker of poor tumour prognosis associated with reduced survival. A meta-analysis [59] studied the relative frequency of CDH1 gene methylation—encoding E-cadherin—in oral cancer, demonstrating that this epigenetic mechanism may be involved in the loss of expression of this relevant adhesion molecule. One line of evidence points to the scarcity of existing studies on transcription factors involved in EMT [26,35,39,40,75]. In this regard, only a systematic review and meta-analysis [75], performed on a small number of primary level studies, showed that Twist overexpression did not influence the survival of oral cancer patients, although these results are not very robust and perhaps a larger sample size may reveal the actual prognostic influence of this marker.

### 4.6. Enabling Characteristics

Hanahan and Weinberg [2] point out that cancer hallmarks are largely acquired progressively as a consequence of a number of enabling characteristics that provide suitable conditions for the development of cancer hallmarks. Among these, they point to the genome instability of tumour cells and the inflammatory state that underlies most pre-invasive lesions and frankly invasive neoplasms as determinants.

The concept of genome instability refers to the tendency to develop mutations and other genome alterations secondary to possible errors that occur during cell division. As it seems logical to assume, genomic instability becomes progressively more evident in cell populations with an increased and out-of-control proliferative state, which promotes the appearance of genome alterations that will enable the acquisition of cancer hallmarks, which in turn will be clonally transmitted to the altered cell progeny. Increased proliferative activity is therefore the main cause of genome instability and the essential driver of new hallmark acquisition, and is therefore now considered an enabling feature. Genome integrity surveillance mechanisms, which essentially rely on the actions of the tumour suppressor gene TP53, largely prevent—through DNA repair or induction of apoptosis—spontaneous mutations occurring during cell division from spreading to clonal populations and providing the basis for the accumulation of new alterations in the multi-step process of cancer progression. Thus the acquisition of an increased proliferative state and the genome instability that this triggers is essentially due to the failure of the functions of tumour suppressor genes, essentially TP53 [231]. Another important group of genes whose alterations are associated with genomic instability are called caretaker genes, for example, BRCA1 and BRCA2, whose mutations in family groups predispose to the development of breast cancer. Although caretaker genes are different from tumour suppressor genes, they have similar functions related to the repair of damaged DNA [220]. Finally, as previously discussed, the loss of telomeric DNA at each cell cycle, when it reaches extreme stages, unprotects DNA ends resulting in end-to-end fusions, breaks and other alterations that favour gene amplifications and mutations that activate oncogenes or inactivate tumour suppressor genes. The activity of telomerase, an enzyme responsible for maintaining proper telomere length, prevents the occurrence of genomic instability associated with telomere shortening [199]. As previously reflected in this paper, the acquisition of genomic instability associated with telomere shortening is enhanced by TP53 alterations, and the increased telomerase activity in fully established malignant clones is used, perhaps in a designed manner, by tumour cells to their own advantage to increase their survival.

The publication of the human cancer genome atlas has made it possible to identify the most prevalent genomic alterations in different types of human tumours. This report includes an analysis of 279 head and neck carcinomas, of which the majority are oral cavity (62%) and oropharyngeal (12%) carcinomas. A recent study, based on data extracted from the non-meta-analytic human genome atlas of cancer [232], has reported the most frequently mutated genes in these tumours. Thus, in HPV-associated tumours, a predominance of mutations in the PI3CA gene and amplifications in the E2F1 gene has been demonstrated. In smoking-associated carcinomas, mutations with loss of function of TP53 and inactivation of the CDKN2A gene, which encodes the p16 tumour suppressor protein, are almost universally demonstrated, together with frequent copy number alterations, including amplifications of 3p26/28 and 11q13—areas that harbour important oncogenes such as CCND1, which encodes cyclin D1. As can be seen, many of the genome alterations present in oral cancer affect the function of tumour suppressor genes or activate oncogenes whose essential function is linked to increased cell proliferation with the development of genome instability. This report again points to the importance of genomic instability as an enabling feature in oral cavity cancer. However, it should be noted that there are no studies with high-evidence designs (systematic reviews and meta-analyses) that provide information on this enabling feature in this neoplasm.

Another enabling feature noted by Hanahan and Weinberg [2] refers to the inflammation that develops adjacent to the majority of tumours. Initially, this peritumoural inflammation was interpreted as an anti-tumour immune response mechanism. While this is true, it is now known that tumour-associated inflammation plays a paradoxical role in favouring malignant transformation and tumour progression [197,209,233,234]. In oral carcinogenesis, some clinical evidence supports this idea, including the tendency for carcinomas to develop in oral lesions of autoimmune aetiology in which a chronic inflammatory infiltrate consistently underlies juxtaposed to the oral epithelium; the most representative example of such autoimmune disorders is oral lichen planus, now considered an oral potentially malignant disorder [12,235,236,237]. Chronic inflammation may contribute to the acquisition of multiple hallmarks of cancer that contribute to tumour initiation and progression of neoplasia, which it executes through the delivery of different bioactive molecules to the tumour microenvironment, including growth and proangiogenic factors, survival factors, transcription factors that induce EMT, matrix metalloproteinases that facilitate invasion, etc. [197,209,233]. In addition, inflammatory cells can release ROS and other substances—such as COX2—that behave as inducers of mutations in the adjacent epithelium [197,238]. Many of these bioactive molecules produced by inflammatory cells have been previously outlined elsewhere in this paper.

### 4.7. Reprogramming Energetic Metabolism

**Figure 4:**
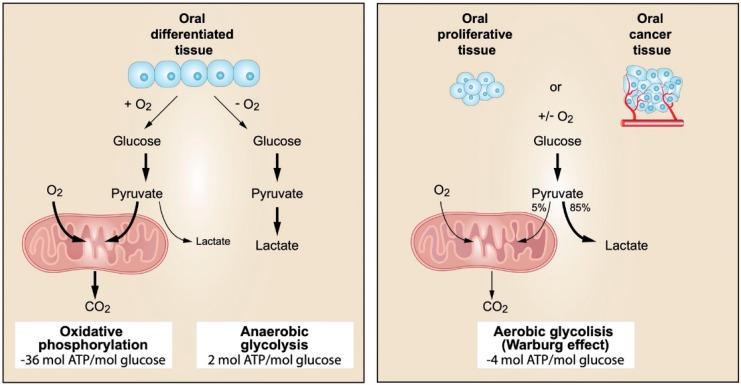
Graphic representation of the reprogramming energetic metabolism in oral squamous cell carcinomas, modified from Vander Heiden et al. [239]. Energetic metabolism in mammalian cells depends essentially, but not exclusively, on the consumption of glucose. The type of energy metabolism adapted by cells is primarily influenced by the availability of oxygen in the surrounding environment and by their proliferative state. Quiescent cells from oral differentiated tissues, in the presence of oxygen, adopt a type of metabolism called oxidative phosphorylation, converting glucose through glycolysis into pyruvate, which is subsequently converted into CO_2_ in the mitochondria through the tricarboxylic acid cycle (TCA). In this process, the cofactor NADH (nicotinamide adenine dinucleotide reduced) is produced, which is relevant for maximising energy production in the form of ATP (adenosine 5’-triphosphate). During oxidative phosphorylation, a small amount of glucose is metabolised to lactate production. The net result of this energy programme is the production of 32 molecules of ATP for each molecule of glucose. In hypoxic situations, quiescent cells develop a type of metabolism known as anaerobic glycolysis, which shifts glucose metabolism towards lactate production, resulting in a much lower energy yield compared to oxidative phosphorylation, resulting in 2 molecules of ATP for each molecule of glucose metabolised. On the other hand, oral proliferative tissues and oral cancer cells develop an apparently paradoxical type of metabolism, called aerobic glycolysis (also known as the Warburg effect), whereby, even in the presence of oxygen, most of the glucose (85%) is derived to produce lactate, while a small percentage (5%) is metabolised in the mitochondria to produce CO_2_ and a small amount of ATP—4 molecules of ATP for each molecule of glucose.

The increased proliferation and sustained cell growth that cancer cells develop impose mandatory modifications of their metabolism to sustain a process that will necessarily consume a large amount of energy and biomass [2]. Cellular energetic metabolism depends essentially, but not exclusively, on the consumption of glucose. The type of energy metabolism in mammalian cells is influenced primarily by the availability of oxygen in the environment and by their proliferative state (Figure 4). In quiescent differentiated cells in the presence of oxygen, a type of metabolism called oxidative phosphorylation takes place, whereby glucose is converted through glycolysis into pyruvate, which is converted into CO_2_ in the mitochondria through the tricarboxylic acid cycle (TCA) with the presence of oxygen. In this process, the cofactor NADH (nicotinamide adenine dinucleotide reduced) is produced, which is relevant for maximising energy production in the form of ATP (adenosine 5’-triphosphate). During oxidative phosphorylation, a small amount of glucose is metabolised to lactate production. The net result of this energy programme is the production of 32 molecules of ATP for each molecule of glucose [239]. In oxygen-deprived situations, quiescent cells develop a type of metabolism called anaerobic glycolysis, which shifts glucose metabolism towards lactate production, resulting in a much lower energy yield compared to oxidative phosphorylation—2 molecules of ATP for each molecule of glucose metabolised. In contrast, proliferative cells and tumour cells develop an apparently paradoxical type of metabolism, called aerobic glycolysis, whereby, even in the presence of oxygen, most of the glucose (85%) is derived to produce lactate, while a small percentage (5%) is metabolised in the mitochondria to produce CO_2_ and a small amount of ATP—four molecules of ATP for each molecule of glucose. It is striking that a proliferative tumour cell, which in theory consumes a large amount of energy, shifts its metabolism to a much less efficient pathway—aerobic glycolysis. Otto Warburg was the first researcher who observed and became interested in 1924 [240,241,242] in this strange, and to this day little explained, metabolic choice of tumour cells, which is known in his honour as the Warburg effect.

What reasons could justify the fact that tumour cells develop a type of metabolism with little capacity to produce energy? We must recognise that there is no universally accepted explanation, although one fact that seems logical is that, despite producing little ATP, aerobic glycolysis is able to provide sufficient energy to maintain cell proliferation in neoplastic cells. There is evidence that ATP is not a limiting condition in proliferative cells developing in nutrient-rich environments, as is often the case in mammals where there is a continuous supply of glucose and other nutrients through the bloodstream. In contrast, neoplastic and proliferative cells have large metabolic requirements that extend beyond ATP needs, as a consequence of the fact that a dividing cell must replicate its whole cellular content, which requires a large amount of biomass in the form of mainly nucleotides, amino acids and lipids. For example, the synthesis of palmitate—an important component of cell membranes—requires 7 molecules of ATP, 16 carbons (from 8 molecules of acetyl CoA—from the breakdown of glucose—and essential for entry into the Krebs cycle) and 28 electrons (from 14 molecules of NADH) [243]. Similar is the case for amino acid and nucleotide synthesis, and for fatty acylo synthesis—an important intermediate in fatty acid synthesis—which consumes 16 carbons. For most mammalian cells in culture, the only two important catabolised molecules are glucose and glutamine, indicating that they supply most of the biomass and free energy needed for cell growth and division. Thus, oxidative phosphorylation that converts most of the glucose into CO_2_ to maximise energy production in the form of ATP runs counter to the biomass needs of a highly proliferative cell. In contrast, through aerobic glycolysis (Warburg effect) a significant proportion of glucose is derived for the production of macromolecular precursors such as acetyl CoA—necessary for the production of fatty acids—glycolytic intermediates to produce non-essential amino acids and ribose to produce nucleotides [243]. Glutamine catabolism provides nitrogen for amino acid synthesis and NADPH. Aerobic glycolysis produces large amounts of lactate that must be expelled from the cytosol. This may appear to be a waste of biomass as it is known that with each molecule of lactate, three carbons are removed. However, there are pathways for the reuse of lactate, and it has also been documented that tumours have cell populations that preferentially use the lactate produced by their neighbours as their main energy source, with the two populations acting symbiotically [243].

Finally, it is interesting to note that the metabolism of proliferating tumour cells is regulated by pathways, essentially PI3K, activated by tyrosine kinase receptors. PI3K activation stimulates glucose uptake and flux through the early stages of glycolysis by regulating glucose transporter expression, enhancing glucose uptake by hexokinase and stimulating phosphofructokinase [239] activity. Conversely, PI3K negatively regulates the last steps of glycolysis by making glycolytic intermediates available for biomass synthesis. It should be recalled that increased proliferative activity is essentially mediated in tumour cells by pathways involving tyrosine kinase receptors, including the PI3K/AKT pathway, and so it appears that tumour cells activate mechanisms that not only increase their rate of proliferation but also provide the metabolic requirements for this to take place successfully.

In relation to the existing evidence on oral carcinogenesis, only five systematic reviews and three meta-analyses have been published [24,63,67,68,91]. Two of these studies focus on GLUT1 overexpression was significantly associated with poor survival, higher T, N+ status, presence of distant metastases, advanced tumour stage and high histological grade. The work of Botha et al. [24], a systematic review without meta-analysis, shows that the glucose transporters GLUT 1 and 3 have received the most attention in the literature with a significant volume of primary level studies, and a trend towards a negative influence of their overexpression on the prognosis of oral cancer. In contrast, GLUT 2, 4, 8 and 13 have received the least attention, with no primary level studies on GLUT 7 and 14 in oral cancer. Three studies [63,67,91] have addressed the relevance of hypoxia-inducible factors (HIFs) in oral carcinogenesis, with HIF1α and HIF2 α overexpression reported to be significantly associated with poor survival ([63,91], respectively).

### 4.8. Evading Tumour Immune Destruction

There is evidence that the organism develops an anti-tumour immune defence, such that neoplasms that were successfully established have somehow managed to overcome this defence barrier. Hanahan and Weinberg [2] have proposed evasion of the immune response as a characteristic hallmark of the tumour cell. All immune cell types have been identified in human tumours and are found in different topographical locations of the neoplasm—in the centre of the tumour mass, at the margin of invasion and in adjacent tertiary lymphatic structures. The location, density and functional orientation of the different immune cell populations in a tumour are known as the “immune context” [244] and their analysis in large samples of tumour tissues has made it possible to determine which of them exert beneficial effects and which have a negative influence on the patient’s prognosis. Intense lymphocytic infiltration has been reported to be associated with a favourable clinical outcome in many tumour types, including HNSCC. In particular, high densities of TCD3, cytotoxic TCD 8 and CD45RO memory T cells are associated with longer overall survival and disease-free survival [245]. In contrast, the role of regulatory T cells—resulting from the activation of TCD4 cells—is contradictory. The initial report on the subject [246] found a negative prognostic effect on ovarian cancer of high intratumoural CT reg densities, which has been ratified in other tumour types [247,248]. Other reports have found no association with prognosis and, finally, in some tumour types, including HNSCC, higher CT reg density is associated with longer survival. The reasons for this contradiction may lie in the lack of specificity of the markers used in the different studies or perhaps in the influence that the tumour phenotype, unique host factors and consequently the diverse microenvironment in which tumours develop may exert on the functions of these cells. It is possible that reg TCs may exert negative prognostic influences when they block effector T cells or positive ones via reduction of chronic peritumoural inflammation and their tumourigenesis-promoting effect [249,250,251,252,253,254,255,256,257]. The functions of NK cells are unique. When the high specificity marker NCR1 is used for detection, high NK density does not seem to be associated with a good prognosis in advanced stages of oncogenesis (results obtained in small cell lung cancer) [258], although it is associated with a good prognosis in early stages (results found in breast cancer) [259]. It has been reported that NK cells in advanced neoplasms have an anergic phenotype, meaning that they are unable to secrete INF-γ and kill tumour cells. This could be caused by the tumour cells themselves, which in their expansion and development can acquire the ability to secrete TGF-β, which keeps NK cells in an anergic state. In the initial phases of the neoplasm, in which the tumour cells have not yet acquired the capacity to secrete TGF-β, NK cells could fully exert their anti-tumour effector functions. The prognostic influence exerted by B cells is not fully elucidated. In experimental mouse models, infiltrating B cells exert a negative prognostic influence, probably due to their ability to produce IL-10 and IgG, which can activate the M2 pro-tumour phenotype in macrophages and promote early stages of carcinogenesis [260]. B cells, which are clonally expanded in some cancers [261], may promote metastasis by converting resting TCD4 cells into reg TCs, which as discussed above suppress the actions of cytotoxic TCs [262]. However, some reports attribute a favourable prognostic value to B-cell infiltration in breast and ovarian cancer [263,264], while in many other tumour types there are no data. Cytokines and chemokines also exert anti-tumour response actions. A study analysing the genes associated with the anti-tumour immune response in colon cancer [265] has shown that the most influential genes are those encoding chemokine production. Especially the high expression of genes encoding CX3CL1, CXCL9 and CXCL10 has been found in carcinomas highly infiltrated by effector TCs, particularly TH1, which show higher overall and disease-free survival. 

A recently documented mechanism of evasion of the anti-tumour immune response is implemented via overexpression of the transmembrane protein PD-L1. Overexpression of this protein by tumour cells induces apoptosis in infiltrating T lymphocytes thus providing resistance to neoplastic T cell-mediated destruction [266]. Our research group has reported in a case series of OSCCs and in a systematic review and meta-analysis an association between PD-L1 overexpression and poor survival [30,267].

In addition to the anti-tumour functions of the exposed autoimmune cells, other evidence points to the role of immune surveillance in cancer protection. Thus, in immunosuppressed individuals, there is an increased incidence of some types of tumours [267], although most of them are associated with oncogenic viruses and here the role of the immune system would be related to the clearance of virus-infected cells. In immunosuppressed genetically modified mice, exposure to chemical carcinogens generates tumours very frequently and rapidly. These tumours show low infiltration by CD8+ T lymphocytes, CD4+Th1 T helper cells and NK cells, all of which are to a greater or lesser extent effectors of the anti-tumour response. Clinical data also provide evidence in this regard. Patients with carcinomas highly infiltrated by cytotoxic T cells and NK cells, such as colon cancer, have a better prognosis than those without strong effector cell infiltration [2]. Our group, in two studies [268,269], has reported that oral carcinomas developed on oral lichen planus and proliferative verrucous leukoplakia have a better prognosis than conventional oral carcinomas, which could be related to the strong inflammatory infiltrate observed in these tumours. Furthermore, organ transplant recipients from theoretically tumour-free donors under immunosuppression occasionally develop tumours from the donor, presumably because these neoplastic cells from the immunocompetent donor would be maintained at bay by their immune system [270]. Finally, in these chronically immunocompromised patients, the clinical epidemiology shows an increased incidence of tumours associated with oncogenic viruses but not of tumours unrelated to viruses. This may be related to the fact that immunocompromised patients may not be markedly deficient in cytotoxic lymphocytes and NK cells and thus retain some immune surveillance capacity. Taken together, these findings suggest that immune surveillance contributes significantly to tumour eradication [270] and that the capacity for immunoevasion is therefore probably a distinctive feature of cancer that nevertheless needs to be studied further. 

## 5. Conclusions

**Figure 5:**
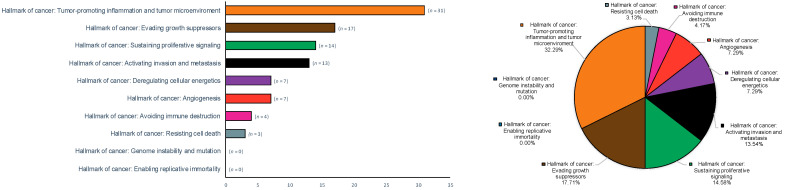
Bar and pie charts graphically summarizing the evidence derived from the research on hallmarks of oral and oropharyngeal carcinogenesis across secondary-level systematic reviews and meta-analyses. Hallmarks of cancer were ordered by absolute counts (**left**, bar chart) and relative frequencies by calculating raw proportions, expressed as percentages (**right**, pie chart).

This scoping review of systematic reviews shows that there is very little evidence-based research, in the form of systematic reviews and meta-analyses, on the prognostic influence of the cancer hallmarks proposed by Hanahan and Weinberg in oral and oropharyngeal carcinogenesis (Figure 5). The complete absence of high-evidence papers on the influence of the Rb gene in oral and oropharyngeal carcinogenesis is striking. There is little information on some tumour suppressor proteins such as p21, p15 or p27. Likewise, there is no information on the mechanisms that allow oral cancer cells to achieve replicative immortality, especially with regard to telomerase overexpression. Otherwise, there is very little information on the mechanisms linked to neoangiogenesis, where one meta-analysis has reported an association between VEGF overexpression and poor OSCC survival, and three other meta-analyses have pointed out the limited impact of VEGF gene polymorphisms on the risk of developing oral cancer. Finally, there is very scarce information on mechanisms related to genomic instability, on the hallmark related to reprogramming of energetic metabolism, where five meta-analyses have a reported poor prognosis of oral cancer associated with overexpression of glucose transporters, and in relation to immune evasion, where one meta-analysis has documented poor prognosis associated with PD-L1 overexpression.

It seems clear that extensive research efforts are required to increase both the number of primary-level studies and the number of systematic reviews and meta-analyses that make it possible to obtain evidence-based results on the influence of different cancer hallmarks in oral and oropharyngeal cancers.

## Figures and Tables

**Table 1:** Summarized study characteristics.

Total Sample	85 Studies
Year of publication	
Range Min (first publication)	2010
Range Max	2022
Study design	
systematic review	16
systematic review and meta-analysis	69
Study population	
oral potentially malignant disorders	8
oral cancer	70
oropharyngeal cancer	3
oral and oropharyngeal cancers	4

**Table 2:** Summarized evidence derived from the research on hallmarks of oral and oropharyngeal carcinogenesis across systematic reviews.

Hallmark of Cancer: Sustaining Proliferative Signalling	
EGFR	2 studies
ERBB2	1 study
Akt	1 study
mTor	1 study
p7056k	1 study
EIF4EBP1	1 study
Stat3	1 study
Cyclin D1	6 studies
**Hallmark of cancer: Evading growth suppressors**	
p53	10 studies
p16	6 studies
p27	1 study
**Hallmark of cancer: Resisting cell death**	
Fas	1 study
FASLG	1 study
FADD	1 study
**Hallmark of cancer: Enabling replicative immortality**	
no evidences	0 studies
**Hallmark of cancer: Angiogenesis**	
VEGF	4 studies
FGFR	1 study
THBS1	1 study
Perycites	1 study
**Hallmark of cancer: Activating invasion and metastasis**	
E-cadherin	4 studies
β-Catenin	1 study
Twist	2 studies
SNAI1	1 study
SNAI2	1 study
Zeb1	1 study
EMT phenomenon	3 studies
**Hallmark of cancer: Deregulating cellular energetics**	
HIF-1α	3 studies
HIF-2α	2 studies
GLUT-1	1 study
GLUTs	1 study
**Hallmark of cancer: Avoiding immune destruction**	
PD-L1	1 study
TILs	1 study
Tregs	1 study
Immune chekpoints	1 study
**Hallmark of cancer: Genome instability and mutation**	
no evidences	0 studies
**Hallmark of cancer: Tumour-promoting inflammation and tumour microenviroment**	
cancer stem cells	3 studies
CD44	1 study
ALDH1	1 study
Interleukins	11 studies
CAFs	3 studies
Macrophages	2 studies
Lymphocytes ratios	3 studies
MMPs	7 studies

note: —More than one biomarker was analysed per study.

**Table 3:** Systematic reviews and meta-analyses published (n = 85).

(A) Systematic Reviews and Meta-Analyses Published in Oral Squamous Cell Carcinoma (OSCC) and Oropharyngeal Squamous Cell Carcinoma (OPSCC)					
Hallmark: Sustaining Proliferative Signalling					
Biomarker	Study	Year	Population	Design	Key Results
EGFR	Perisanidis et al.	2017	OSCC	SR	Seven primary level-studies investigated the presence of *EGFR* mutation status in oral cancer. Only 2 out of 486 cases (0.41%) harboured *EGFR* mutations.
	Marques et al.	2016	OSCC	SR + MTA	Only one primary-level study investigated the prognostic significance of EGFR overexpression in OSCC, reporting a significant association with poor survival (HR = 1.98, 95% CI = 1.01–3.87).
ERBB2	Meng et al.	2020	OSCC	SR + MTA	ERBB2 overexpression was significantly associated with worse overall survival (HR = 2.40, 95% CI = 1.53–2.55), worse disease-specific survival (HR = 2.60, 95% CI = 1.11–4.1), worse disease-free survival (HR = 2.22, 95% CI = 1.46–2.99), N+ satus (OR = 2.23, 95% CI = 1.47–3.36), and advanced clinical stage (OR = 1.84, 95% CI = 1.17–2.88) in OSCC.
Akt	Marques et al.	2016	OSCC	SR + MTA	Only four primary-level studies investigated the prognostic significance of Akt overexpression in OSCC, reporting heterogeneous metrics and high effect sizes (HR ≈ 2)
mTor	Marques et al.	2016	OSCC	SR + MTA	Only one primary-level study investigated the prognostic significance of mTor overexpression in OSCC, reporting a significant association with poor survival (HR = 2.20, 95% CI = 1.13–4.76).
p7056k	Marques et al.	2016	OSCC	SR + MTA	No evidences. No primary level studies were found researching the prognostic role of p7056k overexpression in OSCC.
EIF4EBP1	Marques et al.	2016	OSCC	SR + MTA	No evidences. No primary level studies were found researching the prognostic role of EIF4EBP1 overexpression in OSCC.
Stat3	Wu et al.	2015	OSCC	SR + MTA	Only one primary-level study investigated the prognostic significance of Stat3 overexpression in OSCC, reporting a significant association with poor survival (HR = 5.31, 95% CI = 2.56–11.00).
Cyclin D1	Ramos-Garcia et al.	2018	OSCC	SR + MTA	Cyclin D1 overexpression was significantly associated with worse overall survival (HR = 2.00, 95% CI = 1.59–2.51), worse disease-free survival (HR = 1.46, 95% CI = 1.13–1.87), higher T status (OR = 1.51, 95% CI = 1.07–2.13), N+ status (OR = 2.16, 95% CI = 1.60–2.92), advanced clinical stage (OR = 1.44, 95% CI = 1.15–1.81), and higher histological grade (OR = 1.60, 95% CI = 1.12–2.29) in OSCC.
	Noorlag et al.	2015	OSCC	SR + MTA	Cyclin D1 overexpression was significantly associated with N+ status (OR = 1.95, 95% CI = 1.41–2.70) in OSCC.
	Zhao et al.	2014	OSCC	SR + MTA	Cyclin D1 overexpression was significantly associated with worse overall survival (HR = 1.90, 95% CI = 1.58–2.28), higher T status (OR = 1.62, 95% CI = 1.05–2.50), N+ status (OR = 2.04, 95% CI = 1.57–2.64), advanced clinical stage (OR = 1.52, 95% CI = 1.14–2.02), and higher histological grade (OR = 1.98, 95% CI = 1.36–2.87) in OSCC.
	Wang et al.	2014	OSCC	SR + MTA	CCND1/cyclin D1 A/G870 polymorphism was significantly more frequent in OSCC than in controls.
	Wang et al.	2013	OSCC	SR + MTA	CCND1/cyclin D1 A/G870 polymorphism was not significantly more frequent in OSCC than in controls.
**Hallmark: Evading growth suppressors**					
**Biomarker**	**Study**	**Year**	**Population**	**Design**	**Key results**
p53	Mulder et al.	2021	OPSCC	SR + MTA	TP53 mutations were significantly more frequent in smokers patients with OPSCC. However, no significant differences were observed between drinkers and non-drinkers.
	Sun et al.	2018	OSCC	SR + MTA	TP53 72P/A polymorphism was not significantly more frequent in OSCC than in controls.
	Lin et al.	2018	OSCC	SR + MTA	TP53 72P/A polymorphism was not significantly more frequent in OSCC than in controls.
	Yang et al.	2016	OSCC	SR + MTA	Serum p53 harboured potential diagnostic value with relatively high sensitivity and specificity for OSCC, compared with healthy controls.
	Xian-Tao et al.	2014	OSCC	SR + MTA	TP53 72P/A polymorphism was not significantly more frequent in OSCC than in controls.
	Hou et al.	2015	OSCC	SR + MTA	TP53 72P/A polymorphism was not significantly more frequent in OSCC than in controls.
	Jiang et al.	2013	OSCC	SR + MTA	TP53 72P/A polymorphism was not significantly more frequent in OSCC than in controls.
	Yun et al.	2013	OSCC	SR + MTA	TP53 72P/A polymorphism was not significantly more frequent in OSCC than in controls.
	Tandon et al.	2010	OSCC OPSCC	SR + MTA	p53 overexpression was significantly associated with worse overall survival in OSCC (HR = 1.48, 95% CI = 1.03–2.11), and with better disease-free survival in OPSCC (HR = 0.45, 95% CI = 0.27–0.73).
p16	Mulder et al.	2021	OPSCC	SR + MTA	p16 overexpression was significantly more frequent in non-smokers and non-drinkers patients with OPSCC.
	Qu et al.	2019	OSCC	SR + MTA	CDKN2A/p16 G/C915 polymorphism was significantly more frequent in OSCC than in controls. However, no significant differences were observed for T/C869 and C/T509 polymorphisms.
	Smitha et al.	2017	OSCC	SR + MTA	The overexpression of p16 was assessed across patients with OSCC, showing a pooled proportion of 25.3% (95% CI = 14.3–38.3).
	Sedghizadeh et al.	2016	OPSCC	SR + MTA	p16 overexpression was significantly associated with better overall survival (HR = 0.36, 95% CI = 0.26–0.50) in OPSCC.
	Ndiaye et al.	2014	OSCC OPSCC	SR + MTA	The overexpression of p16 was assessed across patients with positive-HPV OSCC and OPSCC, showing, respectively, pooled proportions of 28.1% (95% CI = 14.3–38.3) and 86.7% (95% CI = 79.2–92.9).
	Don et al.	2014	OSCC	SR + MTA	The promoter hypermethylation of *CDKN2A* was assessed across OSCC patients, showing a pooled proportion of 43% (95% CI = 40–46).
p27	Gao et al.	2013	OSCC	SR + MTA	p27 low expression was significantly associated with N status (RR = 0.59, 95% CI = 0.45–0.79), advanced clinical stage (RR = 1.58, 95% CI = 1.35–1.84), and higher histological grade (RR = 1.35, 95% CI = 1.04–1.77).
**Hallmark: Resisting cell death**					
**Biomarker**	**Study**	**Year**	**Population**	**Design**	**Key results**
Fas	Zhang et al.	2018	OSCC	SR + MTA	Fas -670 A/G and -1377 G/A polymorphisms were not significantly more frequent in OSCC than in controls.
FASLG	Zhang et al.	2018	OSCC	SR + MTA	FASLG -844C/T polymorphism was significantly less frequent in OSCC than in controls.
FADD	Gonzalez-Moles et al.	2021	OSCC	SR + MTA	*FADD* upregulation was significantly associated with poor survival (HR = 1.39, 95% CI = 1.03–1.87) in patients with OSCC.
**Hallmark: Enabling replicative immortality**					
**Biomarker**	**Study**	**Year**	**Population**	**Design**	**Key results**
—	—	—	—	—	—
**Hallmark: Angiogenesis**					
**Biomarker**	**Study**	**Year**	**Population**	**Design**	**Key results**
VEGF	Metzger et al.	2014	OSCC	SR + MTA	VEGF polymorphisms (-2578C/A, -460C/T, 405C/G, -1154G/A and 936C/T) were not significantly more frequent in OSCC than in controls.
	Zhao et al.	2013	OSCC	SR + MTA	*VEGF overexpression* was significantly associated with poor survival (HR = 1.77, 95% CI = 1.09–1.44) in patients with OSCC.
	Kumar et al.	2013	OSCC	SR + MTA	VEGF 936C/T polymorphism was significantly more frequent in OSCC than in controls.
	Zhao et al.	2013	OSCC	SR + MTA	VEGF 936C/T and -460C/T polymorphisms were not significantly more frequent in OSCC than in controls.
FGFR	Ipenburg et al.	2016	OSCC	SR	FGFR1 overexpression was not significantly associated with poor survival in OSCC.
THBS1	Sun et al.	2020	OSCC	SR + MTA	A single primary-level study reported that THBS1 overexpression was significantly associated with better survival in OSCC.
Perycites	Bittencourt et al.	2020	OSCC	SR	The limited data available do not allow to conclude the oncogenic implications of perycites in oral carcinogenesis.
**Hallmark: Activating invasion and metastasis**					
**Biomarker**	**Study**	**Year**	**Population**	**Design**	**Key results**
E-cadherin	de França et al.	2021	OPSCC	SR + MTA	E-cadherin low expression was significantly associated with poor survival in patients with OPSCC.
	Wen et al.	2018	OSCC	SR + MTA	CDH1/E-cadherin hypermethylation was significantly more frequent in OSCC than in controls.
	Luo et al.	2014	OSCC	SR + MTA	E-cadherin low expression was significantly associated with poor survival (HR = 1.54, 95% CI = 1.25–1.92) in patients with OSCC.
	Zhao et al.	2012	OSCC	SR + MTA	E-cadherin low expression was significantly associated with poor survival in patients with OSCC.
β-Catenin	Ramos-Garcia et al.	2021	OSCC	SR + MTA	β-Catenin aberrant expression was significantly associated with worse overall survival (HR = 1.77, 95% CI = 1.20–2.60), worse disease-free survival (HR = 2.44, 95% CI = 1.10–5.50), higher T status (OR = 1.76, 95% CI = 1.23–2.53), N+ status (OR = 2.39, 95% CI = 1.68–3.40), advanced clinical stage (OR = 2.40, 95% CI = 1.58–3.63), and higher histological grade (OR = 1.57, 95% CI = 1.09–2.25) in OSCC.
Twist	Zhou et al.	2015	OSCC	SR + MTA	Twist overexpression was significantly associated with poor survival in patients with OSCC.
	Wan et al.	2019	OSCC	SR + MTA	Twist overexpression was significantly associated with poor survival (HR = 1.61, 95% CI = 1.29–2.02) in patients with OSCC.
SNAI1	Wan et al.	2019	OSCC	SR + MTA	SNAI1 overexpression was significantly associated with poor survival (HR = 2.17, 95% CI = 1.63–2.88) in patients with OSCC.
SNAI2	Wan et al.	2019	OSCC	SR + MTA	SNAI2 overexpression was significantly associated with poor survival (HR = 1.90, 95% CI = 1.38–2.62) in patients with OSCC.
Zeb1	Wan et al.	2019	OSCC	SR + MTA	Zeb1 overexpression was significantly associated with poor survival (HR = 2.70, 95% CI = 1.61–4.53) in patients with OSCC.
EMT phenomenon	Vallina et al.	2021	OSCC	SR	Upregulation of the genes HNRNPC, ITGA5, HMGA2 and SRSF3, and ALDH3A1 and ARID2 downregulation could promote EMT in OSCC
	de Morais et al.	2020	OSCC	SR	EMT biomarkers could harbour oncogenic implications in oral cancer.
**Hallmark: Deregulating cellular energetics**					
**Biomarker**	**Study**	**Year**	**Population**	**Design**	**Key results**
HIF-1α	Zhou et al.	2017	OSCC	SR + MTA	HIF-1α overexpression was significantly associated with worse overall survival (HR = 1.70, 95% CI = 1.10–2.61), higher T status (OR = 2.28, 95% CI = 1.49–3.50), N+ status (OR = 2.05, 95% CI = 1.19–3.53), and advanced clinical stage (OR = 2.29, 95% CI = 1.50–3.49) in OSCC.
	Qian et al.	2016	OSCC	SR + MTA	HIF-1α overexpression was not significantly associated with poor survival in patients with OSCC.
	Gong et al.	2013	OSCC OPSCC	SR + MTA	HIF-1α overexpression was significantly associated with poor survival in patients with OSCC (HR = 2.10, 95% CI = 1.11–3.97) and with OPSCC (HR = 1.76, 95% CI = 1.05–2.97).
HIF-2α	Qian et al.	2016	OSCC	SR + MTA	HIF-2α overexpression was not significantly associated with poor survival in patients with OSCC.
	Gong et al.	2013	OSCC	SR + MTA	HIF-2α overexpression was significantly associated with poor survival (HR = 1.50, 95% CI = 1.02–2.20) in patients with OSCC.
GLUT-1	Li et al.	2016	OSCC	SR + MTA	GLUT-1 overexpression was significantly associated with worse overall survival (HR = 1.88, 95% CI = 1.51–2.33), higher T status (OR = 3.36, 95% CI = 2.04–5.51), N+ status (OR = 3.15, 95% CI = 1.89–5.25), advanced clinical stage (OR = 2.99, 95% CI = 2.01–4.46), and higher histological grade (OR = 3.34, 95% CI = 1.12–9.94) in OSCC.
GLUTs	Botha et al.	2016	OSCC	SR	GLUT-1 and GLUT-3 have been the most investigated GLUTs in oral carcinogenesis, with potential oncogenic implications. While there is insufficient data for GLUTs-2/4/8/13 and no studies for GLUT-7 and GLUT-14.
**Hallmark: Avoiding immune destruction**					
**Biomarker**	**Study**	**Year**	**Population**	**Design**	**Key results**
PD-L1	Lenouvel et al.	2020	OSCC	SR + MTA	PD-L1 overexpression was significantly associated with worse disease-specific survival (HR = 1.74, 95% CI = 1.14–2.66), disease-free survival (HR = 1.56, 95% CI = 1.16–2.09), and advanced clinical stage (OR = 1.63, 95% CI = 1.00–2.64) in OSCC.
TILs	Huang et al.-	2019	OSCC	SR + MTA	Tumour-infiltrating lymphocytes (TILs) biomarkers overexpression (i.e., CD8+, CD45RO+, and CD57+) was significantly associated with better prognosis in patients with OSCC.
Tregs	O’Higgins et al.	2018	OSCC OPSCC	SR	Among recruiting regulatory CD4 T cells (Tregs), FoxP3 has been the most investigated biomarker in oral carcinogenesis, with potential prognostic implications. The lack of knowledge on the role of Tregs in OPSCC is emphasised.
Immune chekpoints	Sieviläinen et al.	2018	OSCC	SR	Seven immune checkpoints (i.e., FKBP51, B7-H4, B7-H6, ALHD1, PD-L1, B7-H3 and IDO1) were found to be associated with poor prognosis in patients with OSCC.
**Hallmark: Genome instability and mutation**					
**Biomarker**	**Study**	**Year**	**Population**	**Design**	**Key results**
—	—	—	—	—	—
**Hallmark: Tumour-promoting inflammation and tumour microenviroment**					
**Biomarker**	**Study**	**Year**	**Population**	**Design**	**Key results**
Cancer stem cells	Singh et al.	2021	OSCC	SR	Among cancer stem cells biomarkers, CD44, ALDH1, and CD133 have been the most expressed in head and neck cancer, including OSCC, with potential oncogenic implications.
	Curtarelli et al.	2018	OSCC	SR	Among cancer stem cells biomarkers, ALDH1, Sox2, Oct4, ABCB5, AGR2 and TAZ were found to harbour oncogenic implications in patients with head and neck carcinomas, including OSCC.
CD44	Chen et al.	2014	OSCC	SR + MTA	CD44 overexpression was not significantly associated with poor survival in patients with OSCC.
ALDH1	Zhou et al.	2014	OSCC	SR + MTA	ALDH1 overexpression was not significantly associated with poor survival in patients with OSCC.
Interleukins	Rezaei et al.	2021	OSCC	SR + MTA	IL-8 (-251T/A) and IL-6 (-174G/C) polymorphisms were not significantly more frequent in OSCC than in controls.
	Liu et al.	2021	OSCC	SR + MTA	IL-10 -1082A/G polymorphism was significantly more frequent in OSCC than in controls.
	Ferrari et al.	2021	OSCC	SR + MTA	Salivary IL-6, IL-8, and TNF-α were significantly more frequent in OSCC than in controls.
	Rezaei et al.	2020	OSCC	SR + MTA	IL-10 -1082A/G polymorphism was significantly more frequent in OSCC than in controls, while IL-10 -592A/C and -819C/T polymorphisms were not.
	Li et al.	2020	OSCC	SR + MTA	IL-10 -1082A/G polymorphism was significantly more frequent in OSCC than in controls.
	Rezaei et al.	2019	OSCC	SR + MTA	Serum and salivary IL-6 and IL-8 were significantly more frequent in OSCC than in controls.
	Yang et al.	2014	OSCC	SR + MTA	IL-8 -251T/A polymorphism was not significantly more frequent in OSCC than in controls.
	Wang et al.	2013	OSCC	SR + MTA	IL-8 -251T/A polymorphism was significantly more frequent in OSCC than in controls.
	Chen et al.	2013	OSCC	SR + MTA	TNF-α 238G/A and 308G/A polymorphisms were significantly more frequent in OSCC than in controls.
CAFs	Knops et al.	2020	OSCC	SR + MTA	An increased cancer-associated fibroblast (CAFs) density was significantly associated with poor prognosis in head and neck squamous cell carcinomas and in OSCC.
	Dourado et al.	2018	OSCC	SR + MTA	The presence of high levels of cancer-associated fibroblast (CAFs) in the stroma was significantly associated with poor overall survival (HR = 2.16, 95% CI = 1.60–2.92) and disease-free survival (HR = 3.32, 95% CI = 2.09–5.26) in OSCC.
Macrophages	Troiano et al.	2019	OSCC	SR + MTA	CD163+ was significantly associated with poor prognosis in patients with OSCC (overall survival: HR = 2.26, 95% CI = 1.47–3.47; progression-free survival: HR = 2.29, 95% CI = 1.11–4.71), while CD68+ was not.
	Alves et al.	2018	OSCC	SR	Among macrophages biomarkers, CD68+, and CD163+ were found to be the most investigated proteins potentially harbouring oncogenic implications in patients with OSCC.
Lymphocytes ratios	Kumarasamy et al.	2021	OSCC	SR + MTA	The higher platelet-lymphocyte, neutrophil-lymphocyte and monocyte-lymphocyte ratios were significantly associated with poor survival in patients with OSCC.
	Yang et al.	2018	OSCC	SR + MTA	The higher neutrophil-lymphocyte ratio was significantly associated with poor overall survival (HR = 1.61, 95% CI = 1.39–1.86), poor disease-free survival (HR = 1.73, 95% CI = 1.44–2.07, higher T status (OR = 3.22, 95% CI = 2.59–4.01), N+ status (OR = 1.62, 95% CI = 1.32–1.98), advanced clinical stage (OR = 2.63, 95% CI = 2.12–3.25) and higher histological grade (OR = 1.48, 95% CI = 1.03–2.11) in patients with OSCC.
	Wang et al.	2018	OSCC	SR + MTA	The higher neutrophil-lymphocyte ratio was significantly associated with poor overall survival (HR = 1.56, 95% CI = 1.28–1.90) and poor disease-specific survival (HR = 1.93, 95% CI = 1.47–2.54) in patients with OSCC.
MMPs	Rezaei et al.	2021	OSCC	SR + MTA	Serum MMP-7 and MMP-9 and salivary MMP-1 and MMP-9 levels were significantly more frequent in OSCC than in controls.
	Miguel et al.	2020	OSCC	SR + MTA	MMPs-1,-2,-3.-7,-9 overexpression were significantly associated with N+ status in OSCC.
	AlAli et al.	2020	OSCC	SR	Salivary MMP-9 harboured potential diagnostic value with relatively high sensitivity and specificity for OSCC, compared with healthy controls.
	Deng et al.	2019	OSCC	SR + MTA	MMPs-2,-9 overexpression were significantly associated with poor survival (HR = 3.93, 95% CI = 2.19–7.07 and HR = 1.96, 95% CI = 1.55–2.48, repectively) in OSCC
	Li et al.	2018	OSCC	SR + MTA	MMP-1 -1607 1G/2G polymorphism was significantly more frequent in OSCC than in controls, while MMP-2 -1306C/T and MMP-3 -11715A/6A polymorphisms were not.
	Zheng et al.	2015	OSCC	SR + MTA	MMP-9 overexpression was significantly associated with poor survival in OSCC.
**(B) Systematic reviews and meta-analyses published in oral potentially malignant disorders (OPMD)**					
**Hallmark: Sustaining proliferative signalling**					
**Biomarker**	**Study**	**Year**	**Population**	**Design**	**Key results**
Cyclin D1	Ramos-Garcia et al.	2019	OPMD	SR + MTA	*CCND1*/cyclin D1 upregulation was significantly associated with higher malignant transformation risk of OPMD (RR = 2.31, 95% CI = 1.46–3.64)
**Hallmark: Evading growth suppressors**					
**Biomarker**	**Study**	**Year**	**Population**	**Design**	**Key results**
p53	Ramos-Garcia et al.	2022	OPMD	SR + MTAA	p53 overexpression was significantly associated with higher malignant transformation risk of OPMD (RR = 1.9, 95% CI = 1.4–2.6), singularly oral leukoplakias (RR = 2.2, 95% CI = 1.4–3.6).
**Hallmark: Resisting cell death**					
**Biomarker**	**Study**	**Year**	**Population**	**Design**	**Key results**
—	—	—	—	—	—
**Hallmark: Enabling replicative immortality**					
**Biomarker**	**Study**	**Year**	**Population**	**Design**	**Key results**
—	—	—	—	—	—
**Hallmark: Angiogenesis**					
**Biomarker**	**Study**	**Year**	**Population**	**Design**	**Key results**
—	—	—	—	—	—
**Hallmark: Activating invasion and metastasis**					
**Biomarker**	**Study**	**Year**	**Population**	**Design**	**Key results**
EMT phenomenon	de Morais et al.	2020	OPMD	SR	EMT biomarkers could be useful to predict the malignant transformation of oral potentially malignant disorders
**Hallmark: Deregulating cellular energetics**					
**Biomarker**	**Study**	**Year**	**Population**	**Design**	**Key results**
—	—	—	—	—	—
**Hallmark: Avoiding immune destruction**					
**Biomarker**	**Study**	**Year**	**Population**	**Design**	**Key results**
—	—	—	—	—	—
**Hallmark: Genome instability and mutation**					
**Biomarker**	**Study**	**Year**	**Population**	**Design**	**Key results**
—	—	—	—	—	—
**Hallmark: Tumour-promoting inflammation and tumour microenviroment**					
**Biomarker**	**Study**	**Year**	**Population**	**Design**	**Key results**
Cancer stem cells	Singh et al.	2019	OPMD	SR + MTA	Among cancer stem cells biomarkers, ABCG2, ALDH1, Bmi1, CD133 and podoplanin have been significantly associated with higher malignant transformation risk in patients with OPMD.
Interleukins	Chiamulera et al.	2021	OPMD	SR + MTA	Salivary IL-6 and TNF-α were significantly more frequent in OPMD than in controls.
	Mehrbani et al.	2020	OPMD	SR	Serum and salivary IL-4 could harbour a role in the development of oral lichen planus.
CAFs	Coletta et al.	2018	OPMD	SR	The immunodetection of cancer-associated fibroblast (CAFs) could be useful to predict the malignant transformation of oral submucous fibrosis.
MMPs	Venugopal et al.	2016	OPMD	SR	Serum, salivary and tisular MMP9 levels were significantly more frequent in OPMD than in controls.

## Appendix A. Search Strategy

MEDLINE/PubMed (*n* = 621)

(“ErbB Receptors”[Mesh] OR “Genes, erbB-1”[Mesh] OR “epidermal growth factor receptor”[all fields] OR “egfr”[all fields] OR erbb*[all fields] OR “EGF Family of Proteins”[Mesh] OR “Epidermal Growth Factor”[Mesh] OR “epidermal growth factor”[all fields] OR “egf”[all fields] OR “Genes, erbB-2”[Mesh] OR “Receptor, ErbB-2”[Mesh] OR “Receptor, ErbB-3”[Mesh] OR “neu”[all fields] OR “erbb2”[all fields] OR “erbb3”[all fields] OR “erbb4”[all fields] OR “cerbb2”[all fields] OR “cerbb3”[all fields] OR “cerbb4”[all fields] OR “her2”[all fields] OR “her3”[all fields] OR “her4”[all fields] OR “cyclin d1”[MeSH] OR (“cyclin”[All Fields] AND “d1”[All Fields]) OR “cyclin d1”[All Fields] OR “cyclind1”[All Fields] OR “ccnd1”[All Fields] OR “ccnd 1”[All Fields] OR “Genes, ras”[Mesh] OR “ras Proteins”[Mesh] OR “ras”[All Fields] OR “hras”[All Fields] OR “kras”[All Fields] OR “nras”[All Fields] OR “Phosphatidylinositol 3-Kinases”[Mesh] OR “pi3k”[All Fields] OR “akt”[All Fields] OR “mtor”[All Fields] OR “pten”[All Fields] OR “NF-kappa B”[Mesh] OR “I-kappa B Kinase”[Mesh] OR “nuclear factor kappa b”[All Fields] OR “nf kappa b”[All Fields] OR “nfkb”[All Fields] OR “i kappa b kinase”[All Fields] OR “ikk”[All Fields] OR “STAT Transcription Factors”[Mesh] OR “Janus Kinases”[Mesh] OR “Signal transducers and activators of transcription”[All Fields] OR “stat”[All Fields] OR “stat3”[All Fields] OR “stat5”[All Fields] OR “janus”[All Fields] OR “jak”[All Fields] OR “jak1”[All Fields] OR “jak2”[All Fields] OR “Mitogen-Activated Protein Kinase Kinases”[Mesh] OR “MAP Kinase Signaling System”[Mesh] OR “Proto-Oncogene Proteins B-raf”[Mesh] OR “mitogen activated protein kinase”[All Fields] OR “mapk”[All Fields] OR “mapkk”[All Fields] OR “mapkkk”[All Fields] OR “braf”[All Fields] OR “mek”[All Fields] OR “erk”[All Fields] OR “Retinoblastoma[Mesh]” OR “retinoblastoma”[All Fields] OR “rb”[All Fields] OR “prb”[All Fields] OR “osrc”[All Fields] OR “pp110”[All Fields] OR “p105-Rb”[All Fields] OR “ppp1r130”[All Fields] OR “p110-rb1”[All Fields] OR “Cyclin-Dependent Kinase Inhibitor p16”[Mesh] OR “p16”[All Fields] OR “cdkn2a”[All Fields] OR “ink4a”[All Fields] OR “Cyclin-Dependent Kinase Inhibitor p15”[Mesh] OR “p15”[All Fields] OR “cdkn2b”[All Fields] OR “ink4b”[All Fields] OR “Cyclin-Dependent Kinase Inhibitor p18”[Mesh] OR “p18”[All Fields] OR “cdkn2c”[All Fields] OR “ink4c”[All Fields] OR “p19”[All Fields] OR “cdkn2d”[All Fields] OR “ink4d”[All Fields] OR “Cyclin-Dependent Kinase Inhibitor p21”[Mesh] OR “p21”[All Fields] OR “cdkn1a”[All Fields] OR “Cyclin-Dependent Kinase Inhibitor p27”[Mesh] OR “p27”[All Fields] OR “cdkn1b”[All Fields] OR “Cyclin-Dependent Kinase Inhibitor p57”[Mesh] OR “p57”[All Fields] OR “cdkn1c”[All Fields] OR “Tumor Suppressor Protein p53”[MeSH] OR “ Genes, p53”[MeSH] OR “p53”[All Fields] OR “tp53”[All Fields] OR “Neurofibromatosis 2”[MeSH] OR “Neurofibromin 2”[MeSH] OR “neurofibromatosis 2”[All Fields] OR “nf2”[All Fields] OR “Neurofibromin 2”[All Fields] OR “merlin”[All Fields] OR “lkb1”[All Fields] OR “Transforming Growth Factor beta”[Mesh] OR “transforming growth factor beta 1”[All Fields] OR “transforming growth factor beta 2”[All Fields] OR “transforming growth factor beta 3”[All Fields] OR “transforming growth factor beta”[All Fields] OR “tgfb” OR “tgf beta”[All Fields] OR “Caspases”[Mesh] OR “Caspase 1”[Mesh] OR “Caspase 2”[Mesh] OR “Caspase 3”[Mesh] OR “Caspase 6”[Mesh] OR “Caspase 7”[Mesh] OR “Caspase 8”[Mesh] OR “Caspase 9”[Mesh] OR “Caspase 10”[Mesh] OR “Caspase 12”[Mesh] OR “Caspase 14”[Mesh] OR caspase*[All Fields] OR “Genes, bcl-2”[Mesh] OR “Proto-Oncogene Proteins c-bcl-2”[Mesh] OR “bcl-X Protein”[Mesh] OR “bcl-2 Homologous Antagonist-Killer Protein”[Mesh] OR “bcl2”[All Fields] OR “bax“[All Fields] OR “bclx“[All Fields] OR “bak“[All Fields] OR “bclw“[All Fields] OR “mcl1“[All Fields] OR “Cytochrome c Group”[Mesh] OR “cytochrome c”[All Fields] OR “cyt c”[All Fields] OR “cyc”[All Fields] OR “NOXA1”[Mesh] OR “NADPH oxidase activator 1”[Mesh] OR “noxa”[All Fields] OR “nadph oxidase activator 1”[All Fields] OR “puma”[All Fields] OR “bbc3”[All Fields] OR “jfy1”[All Fields] OR “bcl2 homology region 3 bh3 only”[All Fields] OR “bh3 only”[All Fields] OR “bim”[All Fields] OR “Autophagy”[Mesh] OR “autophagy”[All Fields] OR “Beclin-1”[Mesh] OR “beclin-1”[All Fields] OR “becn1”[All Fields] OR “atg6”[All Fields] OR “vps30”[All Fields] OR “Necrosis”[Mesh] OR “necrosis”[All Fields] OR “Telomerase”[Mesh] OR “telomerase”[All Fields] OR “tert”[All Fields] OR “Vascular Endothelial Growth Factors”[Mesh] OR (“vascular”[All Fields] AND “endothelial”[All Fields] AND “growth”[All Fields] AND factor*[All Fields]) OR “vegf” [All Fields] OR “Receptors, Vascular Endothelial Growth Factor”[Mesh] OR (“vascular”[All Fields] AND “endothelial”[All Fields] AND “growth”[All Fields] AND factor*[All Fields] AND receptor*[All Fields]) OR “VEGFR”[All Fields] OR “tsp1”[All Fields] OR “tsp”[All Fields] OR “thrombospondin 1”[All Fields] OR “thbs1”[All Fields] OR “thbs”[All Fields] OR “Fibroblast Growth Factors”[Mesh] OR (“fibroblast”[All Fields] AND “growth”[All Fields] AND factor*[All Fields]) OR “fgf” [All Fields] OR “plasmin”[All Fields] OR “angiostatin” [All Fields] OR “Endostatins”[Mesh] OR endostatin*[All Fields] OR “collagen type 18”[All Fields] OR “Pericytes”[Mesh] OR pericyt*[All Fields] OR “rouget cells”[All Fields] OR “Cadherins”[Mesh] OR “e-cadherin”[All Fields] OR cadherin*[All Fields] OR “cd324”[All Fields] OR “cdh1”[All Fields] OR “n-cadherin”[All Fields] OR “CD325”[All Fields] OR “CDH2”[All Fields] OR “Epithelial-Mesenchymal Transition”[Mesh] OR (“epithelial”[All Fields] AND “mesenchymal”[All Fields] AND “transition”[All Fields]) OR “emt”[All Fields] OR snail*[All Fields] OR “slugh2”[All Fields] OR “sna”[All Fields] OR “snah”[All Fields] OR “slug”[All Fields] OR “slugh1”[All Fields] OR “Twist-Related Protein 1”[Mesh] OR “Twist Transcription Factors”[Mesh] OR twist*[all fields] or “bhlh”[all fields] or “scs”[all fields] or “h-twist”[all fields] or “bpes2”[all fields] or “bhlha38”[all fields] or “crs1”[all fields] OR “zeb1”[all fields] OR “zeb2”[all fields] OR “zinc finger E-box binding homeobox 1”[all fields] OR “zinc finger E-box binding homeobox 2”[all fields] OR “tcf8”[all fields] OR “ppcd3”[all fields] OR “bzp”[all fields] OR “zeb”[all fields] OR “areb6”[all fields] OR “nil-2-a”[all fields] OR “zfhep”[all fields] OR “zfhx1a”[all fields] OR “fecd6”[all fields] OR “zfhx1b”[all fields] OR “kiaa0569”[all fields] OR “sip1”[all fields] OR “caretaker”[all fields] OR brca*[all fields] OR “rad51”[all fields] OR “rnf53”[all fields] OR “brcc1”[all fields] OR “ppp1r53”[all fields] OR “fancs”[all fields] OR “fancd1”[all fields] OR “facd”[all fields] OR “fancd”[all fields] OR “rad51a”[all fields] OR “reca”[all fields] OR “hsrad51”[all fields] OR “hst16930”[all fields] OR “brcc5”[all fields] OR “fancr”[all fields] OR “atm”[all fields] OR “ata”[all fields] OR “atdc”[all fields] OR “atc”[all fields] OR “atd”[all fields] OR “tel1”[all fields] OR “telo1”[all fields] OR “DNA Copy Number Variations”[Mesh] OR “Gene Amplification”[Mesh] OR “Sequence Deletion”[Mesh] OR “Glucose Transporter Type 1”[Mesh] OR “glucose transporter type”[All Fields] OR “glut1”[All Fields] OR “hypoxia inducible factor”[All Fields] OR “hif1a”[All Fields] OR “hif-1alpha”[All Fields] OR “pasd8”[All Fields] OR “bHLHe78”[All Fields] OR “HIF2A”[All Fields] OR “HIF-1 alpha-like factor”[All Fields] OR “mop2”[All Fields] OR “pasd2”[All Fields] OR “hlf”[All Fields] OR “bhLhe73”[All Fields] OR “Warburg Effect, Oncologic”[Mesh] OR “warburg”[All Fields] OR “aerobic glycolysis”[All Fields] OR “Isocitrate Dehydrogenase”[Mesh] OR “isocitrate dehydrogenase1”[All Fields] OR “isocitrate dehydrogenase2”[All Fields] OR “idh”[All Fields] OR “idh1”[All Fields] OR “idh2”[All Fields] OR “Tumor Escape”[Mesh] OR ((“evading”[All Fields] OR “evasion”[All Fields] OR “escape”[All Fields]) AND “immune”[All Fields]) OR “Neoplastic Stem Cells”[Mesh] OR “cancer stem cells”[All Fields] OR “csc”[All Fields] OR “Endothelial Cells”[Mesh] OR endothel*[All Fields] OR “Angiopoietin-1”[Mesh] OR “ang-1”[All Fields] OR “angiopoietin 1”[All Fields] OR “kiaa0003”[All Fields] OR “angpt1”[All Fields] OR “Receptor, TIE-2”[Mesh] OR “tie2”[All Fields] OR “vmcm”[All Fields] OR “vmcm1”[All Fields] OR “cd202b”[All Fields] OR “tek”[All Fields] OR “angiopoietin-1 receptor”[All Fields] OR “Receptors, Platelet-Derived Growth Factor”[Mesh] OR “pdgf”[All Fields] OR “platelet derived growth factor receptor”[All Fields] OR “Cathepsins”[Mesh] OR cathepsin*[All Fields] OR “heparanase”[All Fields] OR hpse*[All Fields] OR “Myeloid-Derived Suppressor Cells”[Mesh] OR mdsc*[All Fields] OR “Matrix Metalloproteinases”[Mesh] OR “matrix metallopeptidase”[All Fields] OR “mmp”[All Fields] OR “mmt”[All Fields] OR “mmp1”[All Fields] OR “mmp2”[All Fields] OR “mmp3”[All Fields] OR “mmp7”[All Fields] OR “mmp8”[All Fields] OR “mmp9”[All Fields] OR “mmp10”[All Fields] OR “mmp11”[All Fields] OR “mmp12”[All Fields] OR “mmp13”[All Fields] OR “mmp14”[All Fields] OR “mmp15”[All Fields] OR “mmp16”[All Fields] OR “mmp17”[All Fields] OR “mmp18”[All Fields] OR “mmp19”[All Fields] OR “mmp20”[All Fields] OR “mmp21”[All Fields] OR “mmp24”[All Fields] OR “mmp25”[All Fields] OR “mmp26”[All Fields] OR “mmp27”[All Fields] OR “mmp28”[All Fields] OR “mmp23B”[All Fields] OR “Chemokines”[Mesh] OR “ccl”[All Fields] OR “cxcl”[All Fields] OR “Cytokines”[Mesh] OR interferon*[All Fields] OR interleukin*[All Fields] OR lymphokines*[All Fields] OR monokine*[All Fields] OR oncostatin*[All Fields] OR osteopontin*[All Fields] OR “tumor necrosis factor”[All Fields] OR “tnf-α”[All Fields] OR “macrophages”[Mesh] OR macrophage*[All Fields] OR monocyte*[All Fields] OR histiocyte*[All Fields] OR “Neutrophils”[Mesh] OR neutrophil*[All Fields] OR “polymorphonuclear”[All Fields] OR histiocyte*[All Fields] OR “Cancer-Associated Fibroblasts”[Mesh] OR (“cancer”[All Fields] AND “associated”[All Fields] AND “fibroblasts”[All Fields]) OR “a-sma”[All Fields] OR (“alpha”[All Fields] AND “smooth”[All Fields] AND “muscle”[All Fields] AND “actin”[All Fields]) OR (“stem cells”[All Fields] NOT “cancer stem cells”[All Fields]) OR “hoxa5”[All Fields] OR “homeobox a5”[All Fields] OR “hox1c”[All Fields] OR “hox1”[All Fields] OR “Smad4 Protein”[Mesh] OR “smad4”[All Fields] OR “smad”[All Fields] OR “madh4”[All Fields] OR “mitf”[All Fields] OR “melanocyte inducing transcription factor”[All Fields] OR “ws2a”[All Fields] OR “ws2”[All Fields] OR “bhlhe32”[All Fields] OR “Activating Transcription Factor 2”[Mesh] OR “atf2”[All Fields] OR “activating transcription factor 2”[All Fields] OR “cre-binding protein 1”[All Fields] OR “Retinoic Acid Receptor alpha”[Mesh] OR “rar alpha”[All Fields] OR “rar-a”[All Fields] OR “RUNX1 Translocation Partner 1 Protein”[Mesh] OR runx*[All Fields] OR “aml1-eto”[All Fields] OR “aml1”[All Fields] OR “cbfa2”[All Fields] OR “SOX Transcription Factors”[Mesh] OR “SOXE Transcription Factors”[Mesh] OR “sox10”[All Fields] OR “sry-box transcription factor 10”[All Fields] OR “dom”[All Fields] OR “ws4”[All Fields] OR “ws2e”[All Fields] OR “alpha-ketoglutarate”[All Fields] OR “αkg”[All Fields] OR “a-KG”[All Fields] OR “d-2-hydroxygluterate”[All Fields] OR “d2hg”[All Fields] OR “pancreas associated transcription factor 1a”[All Fields] OR “ptf1a”[All Fields]) AND (“mouth”[MeSH] OR “mouth”[All Fields] OR “oral”[All Fields] OR oropharyn*[All Fields]) AND (“carcinoma, squamous cell”[MeSH] OR (“carcinoma”[All Fields] AND “squamous”[All Fields] AND “cell”[All Fields]) OR “squamous cell carcinoma”[All Fields] OR “Neoplasms”[Mesh] OR neopla*[All Fields] OR “cancer”[All Fields] OR “dysplasia”[All Fields] OR “potentially malignant disorders”[All Fields] OR premalign*[All Fields] OR precancer*[All Fields] OR “leukoplakia”[All Fields] OR “erythroplakia”[All Fields] OR “lichen planus”[All Fields] OR “submucous fibrosis”[All Fields] OR “malignant transformation”[All Fields]) AND (“Meta-Analysis”[pt] OR “meta-analysis”[tiab] OR “Systematic Review”[pt] OR “systematic review”[tiab])

Embase (*n* = 3:853)

(‘epidermal growth factor receptor’/exp OR ‘erbB-1’ OR ‘epidermal growth factor receptor’ OR ‘EGFR’ OR ‘erbb*’ OR ‘epidermal growth factor derivative’/exp OR ‘epidermal growth factor’ OR ‘egf’ OR ‘epidermal growth factor receptor 2’/exp OR ‘erbb2’ OR ‘neu’ OR ‘epidermal growth factor receptor 3’/exp OR ‘erbb3’ OR ‘erbb4’ OR ‘cerbb2’ OR ‘cerbb3’ OR ‘cerbb4’ OR ‘her2’ OR ‘her3’ OR ‘her4’ OR ‘cyclin d1’/exp OR ‘cyclin d1’ OR ‘cyclind1’ OR ‘ccnd1’ OR ‘ccnd 1’ OR ‘ras’ OR ‘hras’ OR ‘kras’ OR ‘nras’ OR ‘Akt signaling’/exp OR ‘pi3k’ OR ‘akt’ OR ‘mtor’ OR ‘pten’ OR ‘NF kB signaling’/exp OR ‘nuclear factor kappa b’ OR ‘nuclear factor kappa b’ OR ‘nf kappa b’ OR ‘nfkb’ OR ‘i kappa b kinase’ OR ‘ikk’ OR ‘JAK-STAT signaling’/exp OR ‘stat’ OR ‘Signal transducers and activators of transcription’ OR ‘stat3’ OR ‘stat5’ OR ‘janus’ OR ‘jak’ OR ‘jak1’ OR ‘jak2’ OR ‘MAPK signaling’/exp OR ‘mitogen activated protein kinase’ OR ‘mapk’ OR ‘mapkk’ OR ‘mapkkk’ OR ‘braf’ OR ‘mek’ OR ‘erk’ OR ‘retinoblastoma’/exp OR ‘rb’ OR ‘prb’ OR ‘osrc’ OR ‘pp110’ OR ‘p105-Rb’ OR ‘PPP1R130’ OR ‘p110-RB1’ OR ‘cyclin dependent kinase inhibitor 2A’/exp OR ‘p16’ OR ‘cdkn2a’ OR ‘ink4a’ OR ‘cyclin dependent kinase inhibitor 2B’/exp OR ‘p16’ OR ‘cdkn2b’ OR ‘ink4b’ OR ‘cyclin dependent kinase inhibitor 2C’/exp OR ‘p18’ OR ‘cdkn2c’ OR ‘ink4c’ OR ‘cyclin dependent kinase inhibitor 2D’/exp OR ‘p19’ OR ‘cdkn2d’ OR ‘ink4d’ OR ‘p21’ OR ‘cdkn1a’ OR ‘p27’ OR ‘cdkn1b’ OR ‘p57’ OR ‘cdkn1c’ OR ‘protein p53’/exp OR ‘p53 signaling’/exp OR ‘p53’ OR ‘TP53’ OR ‘neurofibromatosis type 2’/exp OR ‘merlin’/exp OR ‘neurofibromaatosis 2’ OR ‘nf2’ OR ‘neurofibrin 2’ OR ‘lkb1’ OR ‘transforming growth factor beta’/exp OR ‘transforming growth factor beta 1’ OR ‘transforming growth factor beta 2’ OR ‘transforming growth factor beta 3’ OR ‘transforming growth factor beta 4’ OR ‘ tgfb’ OR ‘ tgf beta’ OR ‘caspase’/exp OR ‘Caspase 1’ OR ‘Caspase 2’ OR ‘Caspase 3’ OR ‘Caspase 6’ OR ‘Caspase 7’ OR ‘Caspase 8’ OR ‘Caspase 9’ OR ‘Caspase 10’ OR ‘Caspase 12’ OR ‘Caspase 14’ OR ‘caspase*’ OR ‘bcl-2’ OR ‘bax’ OR ‘bak’ OR ‘bclw’ OR ‘mcl1’ OR ‘cytochrome c’/exp OR ‘cyt c’ OR ‘cyc’ OR ‘noxa1’ OR ‘NADPH oxidase activator 1’ OR ‘puma’ OR ‘jfy1’ OR ‘bh3’ OR ‘bim’ OR ‘autophagy’ OR ‘beclin-1’ OR ‘becn1’ OR ‘atg6’ OR ‘vps30’ OR ‘necrosis’/exp OR ‘telomerase’/exp OR ‘tert’ OR ‘vasculotropin’/exp OR ‘vegf’ OR ‘vasculotropin receptor’/exp OR ‘vegfr’ OR ‘tsp1’ OR ‘tsp’ OR ‘thrombospondin 1’ OR ‘thbs1’ OR ‘thbs’ OR ‘fibroblast growth factor’/exp OR ‘fgf’ OR ‘plasmin’ OR ‘angiostatin’ OR ‘endostatin’/exp OR ‘collagen type 18’ OR ‘pericyte’/exp OR ‘pericyt*’ OR ‘rouget cells’ OR ‘cadherin’/exp OR ‘e-cadherin’ OR ‘cd324’ OR ‘cdh1’ OR ‘n-cadherin’ OR ‘cd325’ OR ‘cdh2’ OR ‘epithelial mesenchymal transition’/exp OR ‘emt’ OR ‘snail*’ OR ‘slug’ OR ‘slugh1’ OR ‘slugh2’ OR ‘sna’ OR ‘snah’ OR ‘twist’ OR ‘twist*’ OR ‘bhlh’ OR ‘scs’ OR ‘h-twist’ OR ‘bpes2’ OR ‘bhlha38’ OR ‘crs1’ OR ‘zeb1’ OR ‘zeb2’ OR ‘ zinc finger E-box binding homeobox 1’ OR ‘zinc finger E-box binding homeobox 2’ OR ‘tcf8’ OR ‘ppcd3’ OR ‘bzp’ OR ‘zeb’ OR ‘areb6’ OR ‘nil-2-a’ OR ‘zfhep’ OR ‘ zfhx1a’ OR ‘fecd6’ OR ‘zfh1b’ OR ‘kiaa0569’ OR ‘sip1’ OR ‘caretaker’ OR ‘brca*’ OR ‘rad51’ OR ‘rnf53’ OR ‘brcc1’ OR ‘ppp1r53’ OR ‘fancs’ OR ‘fancd1’ OR ‘facd’ OR ‘fancd’ OR ‘rad51a’ OR ‘reca’ OR ‘hsrad51’ OR ‘hst16930’ OR ‘brcc5’ OR ‘fancr’ OR ‘atm’ OR ‘ata’ OR ‘atdc’ OR ‘atc’ OR ‘atd’ OR ‘tel1’ OR ‘telo1’ OR ‘DNA Copy Number Variations’ OR ‘gene amplification’ OR ‘sequence deletion’ OR ‘glucose transporter type 1’ OR ‘glut1’ OR ‘hypoxia inducible factor’ OR ‘hif1a’ OR ‘hif-1alpha’ OR ‘pasd8’ OR ‘bhlhe78’ OR ‘hif2a’ OR ‘hif-1 alpha-like factor’ OR ‘mop2’ OR ‘pasd2’ OR ‘hlf’ OR ‘bhlhe73’ OR ‘warburg’ OR ‘aerobic glycolysis’ OR ‘isocitrate deshydrogenase*’ OR ‘idh’ OR ‘idh1’ OR ‘idh2’ OR ‘tumor escape’ OR ‘immune’ OR ‘stem cell*’ OR ‘csc’ OR ‘endothel*’ OR ‘angiopoietin*’ OR ‘ang-1’ OR ‘kiaa0003’ OR ‘angpt1’ OR ‘tie2’ OR ‘vmcm*’ OR ‘cd202b’ OR ‘tek’ OR ‘ Platelet-Derived Growth Factor’ OR ‘PDGF*’ OR ‘cathepsin*’ OR ‘heparanase’ OR ‘hpse*’ OR ‘Myeloid-Derived Suppressor Cells’ OR ‘mdsc*’ OR ‘matrix metalloproteinase’/exp OR ‘mmp’ OR ‘matrix metallopeptidase’ OR ‘mmt’ OR ‘mmp1’ OR ‘mmp2’ OR ‘mmp3’ OR ‘mmp7’ OR ‘mmp8’ OR ‘mmp9’ OR ‘mmp10’ OR ‘mmp11’ OR ‘mmp12’ OR ‘mmp13’ OR ‘mmp14’ OR ‘mmp15’ OR ‘mmp16’ OR ‘mmp17’ OR ‘mmp18’ OR ‘mmp19’ OR ‘mmp20’ OR ‘mmp21’ OR ‘mmp24’ OR ‘mmp25’ OR ‘mmp26’ OR ‘mmp27’ OR ‘mmp28’ OR ‘mmp23b’ OR ‘chemokine’/exp OR ‘ccl’ OR ‘cxcl’ OR ‘cytokine’/exp OR ‘interferon*’ OR ‘interleukin*’ OR ‘lymphokine*’ OR ‘monokine*’ OR ‘oncostatin*’ OR ‘osteopontin*’ OR ‘tumor necrosis factor’ OR ‘macrophage*’ OR ‘monocyte*’ OR ‘histiocyte’ OR ‘neutrophil*’ OR ‘polymorphonuclear’ OR ‘histiocyte*’ OR ‘cancer associated fibroblast’/exp OR ‘a-sma’ OR ‘alpha smooth muscle actin’ OR ‘stem cell*’ OR ‘hoxa5’ OR ‘homeobox a5’ OR ‘hox1c’ OR ‘hox1’ OR ‘smad*’ OR ‘madh4’ OR ‘mitf’ OR ‘melanocyte inducing transcription factor’ OR ‘ws2a’ OR ‘ws2’ OR ‘bHLHe32’ OR ‘Activating Transcription Factor 2’ OR ‘atf2’ OR ‘CRE-Binding Protein 1’ OR ‘Retinoic Acid Receptor alpha’ OR ‘Rar alpha’ OR ‘rar-a’ OR ‘runx*’ OR ‘AML1-ETO’ OR ‘AML1’ OR ‘CBFA2’ OR ‘sox’ OR ‘soxe’ OR ‘sox10’ OR ‘SRY-box transcription factor 10’ OR ‘dom’ OR ‘ws4’ OR ‘ws2e’ OR ‘alpha-ketoglutarate’ OR ‘αKG’ OR ‘a-KG’ OR ‘D-2-hydroxygluterate’ OR ‘d2hg’ OR ‘pancreas associated transcription factor 1a’ OR ‘PTF1a’) AND (‘mouth’/exp OR ‘mouth’ OR ‘oral’ OR ‘oropharyn*’) AND (‘squamous cell carcinoma’/exp OR ‘carcinoma’ OR ‘malignant neoplasm’/exp OR ‘neoplas*’ OR ‘cancer’ OR ‘dysplasia’ OR ‘potentially malignant disorders’ OR ‘premalign*’ OR ‘precancer’/exp OR ‘precancer*’ OR ‘leukoplakia’/exp OR ‘leukoplakia’ OR ‘erythroplakia’ OR ‘erythroplakia’ OR ‘lichen planus’/exp OR ‘lichen planus’ OR ‘submucous fibrosis’ OR ‘malignant transformation’) AND (‘systematic review’:ti,ab OR [systematic review]/lim OR ‘meta-analysis’:ti,ab OR [meta analysis]/lim)

Cochrane Library (*n* = 24)

((“mouth” OR “oral” OR oropharyn*) AND (“squamous cell carcinoma” OR “dysplasia” OR “potentially malignant disorders” OR “lichen planus” OR “leukoplakia” OR “erythroplakia” OR “submucous fibrosis”)):ti,ab,kw

DARE (*n* = 18)

((“mouth” OR “oral” OR oropharyn*) AND (“squamous cell carcinoma” OR “dysplasia” OR “potentially malignant disorders” OR “lichen planus” OR “leukoplakia” OR “erythroplakia” OR “submucous fibrosis”)):Any field
